# Synthesis, Anti-Inflammatory, and Ulcerogenicity Studies of Novel Substituted and Fused Pyrazolo[3,4-*d*]pyrimidin-4-ones

**DOI:** 10.3797/scipharm.1211-21

**Published:** 2013-01-07

**Authors:** Alaa A. El-Tombary

**Affiliations:** Department of Pharmaceutical Chemistry, Faculty of Pharmacy, University of Alexandria, Alexandria 21521, Egypt.

**Keywords:** Pyrazolo[3,4-*d*]pyrimidin-4-ones, Pyrazolo[3′,4′:4,5]pyrimido[1,2-*b*]pyridazin-4-ones, 7,8,9,10-Tetrahydropyrazolo[3′,4′:4,5]pyrimido[1,2-*b*]cinnolin-4-one, C log P, Synthesis, Anti-inflammatory activity, Ulcerogenicity

## Abstract

In the present investigation, some new pyrazolo[3,4-*d*]pyrimidin-4(5*H*)-one derivatives (**7–12**) as well as fused pyrazolo[3′,4′:4,5]pyrimido[1,2-*b*]pyridazin-4(1*H*)-one (**14–16**) and 7,8,9,10-tetrahydropyrazolo[3′,4′:4,5]pyrimido[1,2-*b*]-cinnolin-4(1*H*)-one (**17**) ring systems were synthesized using the starting compound 5-amino-6-methyl-1-phenyl-1,5-dihydro-4*H*-pyrazolo[3,4-*d*]pyrimidin-4-one (**5**). The structures of the newly synthesized compounds were elucidated by IR, ^1^H NMR, ^13^C NMR, mass spectroscopy, and elemental analysis. The theoretical calculation of their lipophilicity as C log P was performed. The anti-inflammatory activity of all newly synthesized compounds was evaluated using the carrageenan-induced paw edema test in rats using indomethacin as the reference drug. Ulcer indices for the most active compounds were calculated. Seven compounds (**10b**, **11a–f**) showed consistently good anti-inflammatory activity. In particular, 5-{[4-(4-bromophenyl)-3-(4-chlorophenyl)-1,3-thiazol-2(3*H*)-ylidene]amino}-6-methyl-1-phenyl-1,5-dihydro-4*H*-pyrazolo[3,4-*d*]pyrimidin-4-one (**11e**) and its 3,4-bis(4-chlorophenyl) analog (**11f**) were found to be the most effective among the other derivatives, showing activity comparable to that of indomethacin with minimal ulcerogenic effects. Correlation of the biological data of the active compounds with their theoretically calculated C log P values revealed that lipophilicity influences the biological response.

## Introduction

Nonsteroidal anti-inflammatory drugs (NSAIDs) are among the most widely used therapeutics, primarily for the treatment of pain and inflammation in arthritis for decades. NSAIDs reduce the pain and swelling associated with arthritis by blocking the metabolism of arachidonic acid by cyclooxygenase enzyme (COX) thereby the production of prostanoids (including prostaglandins, prostacyclins, and thromboxanes) [1]. Since prostanoids subserve housekeeping functions, such as gastric epithelial cytoprotection and homeostasis besides their known proinflammatory role [2], administration of NSAIDs in the long-term may lead to the development of threatening GI ulcers, bleeding, and renal disorders [3, 4]. Therefore, the discovery of new and safer anti-inflammatory drugs represents a challenging goal for such a research area [5].

**Fig. 1:**
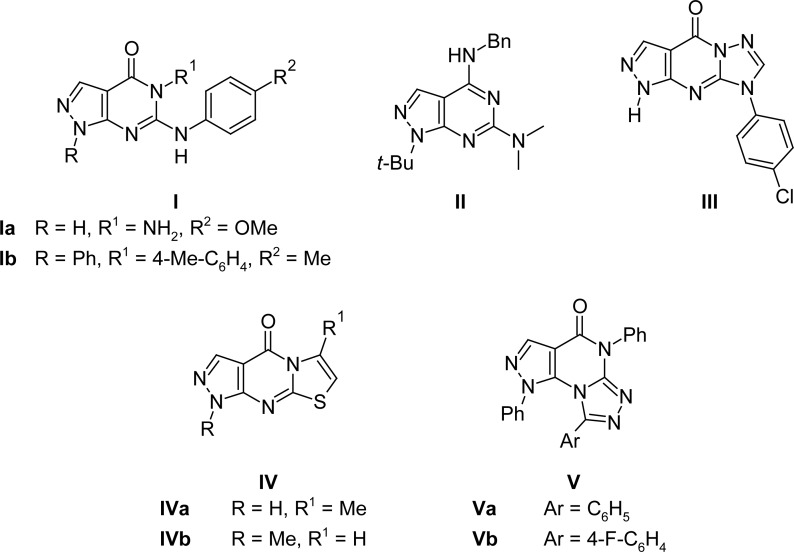
Some selected models of pyrazolopyrimidine derivatives possessing anti-inflammatory activity.

In the course of a research study devoted to the development of new classes of anti-inflammatory drugs, several pyrazolopyrimidine derivatives have been synthesized and have shown potential anti-inflammatory activity associated with remarkable systemic and gastric tolerance. These are exemplified by the variously substituted bicyclic pyrazolo[3,4-*d*]pyrimidine **Ia,b**[6, 7] and **II**[8] derivatives and the tricyclic pyrazolo[3,4-*d*]-[1,2,4]triazolo[1,5-*a*]pyrimidin-4-one **III**[6], pyrazolo[3,4-*d*][1,3]thiazolo[3,2-*a*]pyrimidin-4-one **IVa,b**[9], and pyrazolo[3,4-*e*][1,2,4]triazolo[4,3-*a*]pyrimidin-5-one **Va,b**[10, 11] derivatives (Fig. 1). Among these derivatives, compound **II**[8] inhibited potently cyclooxygenase-2 activity in intact cell assays (IC_50_ = 0.9 nM) with minor activity against cyclooxygenase-1 (IC_50_ = 59.6 nM).

Additionally, a large number of Schiff’s bases [12], thioureas [13], thiazolidinones [14, 15], thiazolines [16], and dioxopyrimidines [17] were reported to possess anti-inflammatory activity.

Since the combination of two pharmacophores on the same scaffold is a well-established approach to the synthesis of more potent drugs [18, 19], it is intended in the present work to incorporate the flexible straight chain azomethine and thiouredo moieties (compounds **7** and **9**) as well as the rigid abovementioned heterocyclic nuclei either directly attached (compounds **8** and **12**) or through imino linkage (compounds **10** and **11**) at the 5-position of the pyrazolo[3,4-*d*]pyrimidine nucleus, hoping that these new hybrids would produce enhanced anti-inflammatory activity.

Furthermore, some heterocyclic compounds containing pyridazine [20, 21] and cinnoline [22] moieties have been recently reported as anti-inflammatory agents. This led to the synthesis of some new heterocyclic compounds containing pyridazine (compounds **14**–**16**) and 5,6,7,8-tetrahydrocinnoline (compound **17**) rings fused with a pyrazolo[3,4-*d*]-pyrimidine nucleus in an effort to synthesize new tricyclic and tetracyclic derivatives to investigate the effect of such a molecular variation on the anti-inflammatory activity.

The enhanced overall lipophilic characteristics of the target compounds could favor their selectivity towards the COX-2 enzyme over COX-1 leading to an increase in the GIT safety margin [23]. Therefore, the substitution pattern of the pharmacophoric groups was selected so as to impart various electronic and lipophilic properties to the molecules, which may contribute to the enhancement of the anti-inflammatory activity of the pyrazolopyrimidine nucleus. In addition to the targeted anti-inflammatory activity, it was also considered of interest to examine the ulcerogenic profiles of the newly synthesized compounds.

## Results and Discussion

### Chemistry

The synthetic pathways adopted for the preparation of the intermediate and target compounds are illustrated in Schemes 1, 2, and 3. The aminoester **2** was prepared following the previously reported procedure [24, 25], by reacting ethyl (ethoxymethylene)cyanoacetate (**1**) [26] with phenylhydrazine (Scheme 1). Hydrolysis of the aminoester **2** with alcoholic sodium hydroxide followed by neutralization afforded the corresponding carboxylic acid **3** which was then refluxed with acetic anhydride to give 6-methyl-1-phenyl-2,3-dihydropyrazolo[3,4-*d*][1,3]oxazin-4(1*H*)-one (**4**). Condensation of the pyrazolooxazine **4** with hydrazine hydrate afforded the key intermediate 5-amino-6-methyl-1-phenyl-1,5-dihydro-4*H*-pyrazolo[3,4-*d*]pyrimidin-4-one (**5**) [27] (Method A). In an alternative route (Method B), compound **5** was prepared by acetylating the aminoester **2** with acetic anhydride followed by reacting the N-acetylated compound **6**[27] with hydrazine hydrate, following the reaction conditions adopted for the synthesis of related compounds [28]. The second route was more advantageous than the first being relatively simple and giving higher reproducible yields of compound **5** (Scheme 1). The IR spectrum of compound **5** showed the NH absorption bands at 3319, 3210, 3112 cm^−1^ together with the amide C=O absorption band at 1695 cm^−1^. Its ^1^H NMR showed the methyl protons and CH-3 as two singlets at δ 2.58 and 8.29 ppm, respectively, beside a D_2_O exchangeable singlet at δ 5.73 ppm integrated for two protons assigned for the NH_2_ group in addition to other aromatic proton signals at their expected chemical shifts. Its ^13^C NMR spectrum showed a highly shielded signal due to the methyl carbon at the 6-position of the pyrazolopyrimidine nucleus at δ 23.21 ppm, and another signal due to C-3a at δ 96.64 ppm. In addition, the spectrum of **5** showed three signals due to the five methine carbons in the aromatic region at δ 122.75, 127.57, and 129.82 ppm indicating the equivalency between the two ortho and the two meta carbons of the phenyl moiety and a signal at δ 136.27 ppm for the phenyl C-1’ quaternary carbon. The spectrum also showed four signals at δ 138.50, 138.72, 159.70, and 164.96 ppm due to C-3, C-7a, C-4, and C-6, respectively.

Condensation of **5** with benzaldehyde or *p*-chlorobenzaldehyde in glacial acetic acid afforded the corresponding 5-(arylideneamino)-6-methyl-1-phenyl-1,5-diyhdro-4*H*-pyrazolo-[3,4-*d*]pyrimidin-4-one derivatives **7a,b** (Scheme 2). The IR spectra of compounds **7a,b** showed disappearance of the absorption bands for the NH_2_ group present in the precursor **5**. Their ^1^H NMR spectra revealed singlet signals resonating at δ 8.35 and 8.88 ppm, respectively, due to the N=CH proton.

Cyclocondensation of Schiff’s bases **7a,b** with thioglycolic acid in refluxing dry benzene, as reported for the preparation of related compounds [29], afforded the corresponding 5-(2-aryl-4-oxo-1,3-thiazolidin-3-yl) derivatives **8a,b**. The IR spectrum of **8b**, as a representative example, showed a new band at 1718 cm^−1^ attributed to thiazolidinone C=O group. Its ^1^H NMR spectrum showed two doublets resonating at δ 3.56 and 3.65 ppm due to the non-equivalent, geminal methylene protons (S-CH_2_) of the 4-thiazolidinone ring interacting with the chiral center at position 2′. The spectra also showed a singlet resonating at δ 5.56 ppm assigned for the methine proton at the 2′-position. Furthermore, the spectrum of **8b** showed a singlet at δ 5.75 ppm integrated for one proton assigned for the enol form of thiazolidinone CH-5′ in addition to a D_2_O exchangeable singlet at δ 9.68 ppm integrated for one proton assigned for the enolic OH proton pointing out that compound **8b** exhibited a typical keto-enol tautomerism. The ^13^C NMR spectrum of compound **8b** showed the signals corresponding to the methylene and the methine carbons C-5′ and C-2′ at δ 62.82 and 63.68 ppm, respectively, and the signal corresponding to the quaternary carbonyl carbon C-4′ at δ 170.37 ppm. The spectrum also showed two signals at δ 90.58 and 179.40 ppm due to enolic C-5′ and C-4′, respectively, confirming the keto-enol tautomerism in the thiazolidinone derivative **8b**. In addition, the spectrum showed signals attributed to the carbons of the 4-chlorophenyl and phenyl substituents at their expected chemical shifts.

Treatment of **5** with phenyl or *p*-chlorophenyl isothiocyanate in refluxing ethanol gave 5-(arylthiocarbamoyl)amino derivatives **9a,b.** The IR spectrum of compounds **9a**, as a representative example, was characterized by the appearance of absorption bands at 1549, 1257, 1220, and 1045 cm^−1^ attributed to N–C=S function [30]. Its ^1^H NMR spectrum showed two deuterium exchangeable singlets due to protons of the aryl-*NH* and pyrazolopyrimidine-*NH* at δ 9.55 and 9.93 ppm. The ^13^C NMR spectrum of **9a** showed, in addition to the signals of the 6-methylpyrazolopyrimidinone and the two phenyl group carbons at their expected chemical shifts, a highly deshielded signal at δ 181.98 ppm due to C=S.

Cyclocondensation of the thiourea derivatives **9a,b** with ethyl bromoacetate, or the appropriate phenacyl bromide in refluxing absolute ethanol, in the presence of anhydrous sodium acetate gave the corresponding 5-[(3-aryl-4-oxo-1,3-thiazolidin-2-ylidene)amino] derivatives **10a,b** or 5-[(3,4-diarylthiazol-2(3*H*)-ylidene)amino] derivatives **11a–f**, respectively. The IR spectra of **10a,b** and **11a–f** lacked the mixed vibrational bands due to N–C=S function present in their precursors **9a,b.** In addition, a new band at 1741 or 1714 cm^−1^ appeared in the IR spectra of **10a,b**, respectively, attributed to the thiazolidinone C=O group. The ^1^H NMR spectra of the products **10b** and **11a**, as representative examples, lacked the NH protons of the parent compounds **9a,b** and showed the singlet at δ 4.03 ppm integrated for two protons due to the thiazolidinone CH-5′ in compound **10b** and the singlet at δ 6.21 ppm integrated for one proton due to thiazoline CH-5′ in compounds **11a**. In addition, the ^1^H NMR spectra of **10b** showed a singlet at δ 6.22 ppm integrated for one proton assigned for the enol form of thiazolidinone CH-5′ in addition to a D_2_O exchangeable singlet at δ 10.68 ppm integrated for one proton assigned for the enolic OH proton. This indicated that compound **10b** would exhibit a typical keto-enol tautomerism. The ^13^C NMR spectrum of **10b** revealed a shielded signal due to the methylene carbon C-5′ at δ 62.35 ppm and two deshielded signals at δ 149.08 and 168.25 ppm due to the quaternary carbons C-2′ and C-4′, respectively. In addition, the ^13^C NMR spectrum of **10b** concluded the presence of keto-enol tautomerism as revealed from the two signals due to the enolic form of thiazolidinone C-5′ and C-4′ resonating at δ 85.88 and 181.17 ppm, respectively. The ^13^C NMR spectrum of the thiazoline **11a** revealed a signal due to the methine carbon of the thiazole C-5′ at δ 88.34 ppm and signals characteristic for the two deshielded quaternary carbons C-4′ and C-2′ at δ 146.55 and 157.04 ppm, respectively. The ^13^C NMR spectra of **10b** and **11a** also showed signals corresponding to other carbons at their expected chemical shifts. Mass spectrum of **11c** revealed its M^+●^+2 and M^+●^ peaks at m/z 512 and 510, respectively, which confirmed its molecular weight. However, molecular ion peaks were not detected in the mass spectra of compounds **11a,b** and **11d–f**.

In addition, the thiourea derivatives **9a,b** were cyclized to 1-Aryl-3-(6-methyl- 4-oxo-1-phenyl-1,4-dihydro-5*H*-pyrazolo[3,4-d]pyrimidin-5-yl)-2-thioxodihydropyrimidine-4,6(1*H*,5*H*)-dione derivatives **12a,b** by reaction with malonic acid in the presence of acetyl chloride following the previously reported reaction conditions [31]. The IR spectrum of **12a**, as a representative example, showed two new absorption bands at 1707 and 1658 cm^−1^ attributed to the dioxopyrimidine C=O groups, besides the absorption band for the pyrazolopyrimidine C=O group, together with mixed vibrational bands due to N–C=S function present in their precursors **9a,b.** Its ^1^H NMR spectrum showed the disappearance of the NH absorption bands of the precursors **9a,b** and the presence of a singlet at 3.75 ppm integrated for two protons assigned for the 4,6-dioxopyrimidine CH-5′. No signal for a vinylic proton was observed in the downfield region, suggesting that compound **12a** exists predominantly in the keto tautomeric form. The ^13^C NMR spectrum of compound **12a** showed a highly shielded signal corresponding to the methylene carbon C-5′ at δ 52.49 ppm beside three signals at 151.78, 152.97, and 157.43 ppm attributed to the quaternary carbons C-4′, C-6′, and C-2′, respectively, in addition to signals due to other carbons resonated at their expected chemical shifts.

On the other hand, oxidation of the 6-methyl group in **5** with selenium dioxide in dioxane following the reported procedure [32] afforded the corresponding 5-amino-4-oxo-1-phenyl-4,5-dihydro-1*H*-pyrazolo[3,4-*d*]pyrimidine-6-carbaldehyde (**13**) (Scheme 3). Its IR spectrum showed characteristic absorption bands at 3441, 3322, and 3188 cm^−1^ corresponding to NH_2_ together with absorption bands at 1720 and 1696 cm^−1^ due to CHO and pyrazolopyrimidine C=O groups, respectively. The ^1^H NMR spectrum of **13** displayed a D_2_O-exchangeable singlet at δ 4.24 ppm integrated for two protons assigned for NH_2_ in addition to the singlet at δ 9.53 ppm characteristic for the CH of the aldehydic group. Its ^13^C-NMR spectrum showed, in addition to the signals corresponding to 4-oxo-1-phenyl-1*H*-pyrazolo[3,4-*d*]pyrimidine carbons at their expected chemical shifts, a highly deshielded signal at δ 172.92 ppm corresponding to the aldehydic group carbon. Its mass spectrum showed a molecular ion peak at m/z 255 which confirmed its molecular weight. Compound **13** was used as a precursor for the synthesis of the tricyclic (**14**–**16**) and tetracyclic (**17**) systems following the reported reaction conditions [33]. Thus, ethyl 7-amino-4-oxo-1-phenyl-1,4-dihydropyrazolo[3′,4′:4,5]pyrimido[1,2-*b*]pyridazine-8-carboxylate (**14**) was prepared by condensing the aminoaldehyde **13** with ethyl cyanoacetate in the presence of sodium ethoxide as a catalyst. The course of the reaction is assumed to involve an intramolecular attack of the amino group on the nitrile function to furnish the target compound **14**.

Likewise, ethyl 7-hydroxy-4-oxo-1-phenyl-1,4-dihydropyrazolo[3′,4′:4,5]pyrimido[1,2-*b*]-pyridazine-8-carboxylate (**15**) was obtained in good yield from **13** and diethyl malonate. The IR spectra of compounds **14** and **15** showed the ester C=O absorption band at 1727 and 1711 cm^−1^, respectively. Compound **14** also showed the NH_2_ absorption bands at 3437, 3287, and 3190 cm^−1^ and the amide C=O at 1678 cm^−1^, whereas compound **15** showed the NH and OH associated absorption bands at 3187–2936 cm^−1^ and the two amide C=O absorption bands at 1649 and 1622 cm^−1^. The ^1^H NMR spectra of compounds **14** and **15** showed three signals corresponding to the protons of the ethyl ester moiety and CH-9 at δ 1.31, 4.15, and 7.68 ppm in the case of compound **14,** and at 1.40, 4.07, and 7.65 ppm in the case of compound **15**, in addition to a D_2_O exchangeable singlet integrated either for two protons at δ 5.06 ppm in the case of compound **14** assigned for NH_2,_ or a half proton at δ 9.60 ppm in the case of compounds **15** assigned for amido-NH.

In addition, the ^1^H NMR spectrum of **15** showed a D_2_O exchangeable singlet at δ 12.33 ppm integrated for a half proton attributed to an imidol-OH. This indicated that compound **15** would exhibit a typical amido-imidol tautomerism. The ^13^C NMR spectrum of **14** showed two high field signals for the ethyl group at δ 15.62 and 62.50 ppm, two signals at δ 129.99 and 143.87 ppm due to the methine carbons C-9 and C-3, respectively, and six signals resonating at δ 87.27, 137.97, 144.16, 145.15, 145.54, and 147.44 ppm corresponding to six quaternary carbons C-3a, C-8, C-10a, C-7, C-4, and C-9a, respectively. The most deshielded signal at δ 148.53 ppm was characterized for the quaternary O-C=O carbon. The spectrum also showed signals corresponding to the phenyl carbons at their expected chemical shifts.

Furthermore, treatment of **13** with acetophenone or cyclohexanone in the presence of sodium ethoxide afforded 1,7-diphenylpyrazolo[3′,4′:4,5]pyrimido[1,2-*b*]pyridazin-4(1*H*)-one (**16**) or 1-phenyl-7,8,9,10-tetrahydropyrazolo[3′,4′:4,5]pyrimido[1,2-*b*]cinnolin-4(1*H*)-one (**17**), respectively, in moderate yield. The ^1^H NMR spectra of compounds **16** and **17** lacked the NH_2_ signal of the precursor **13**. The ^1^H NMR spectrum of compound **16** showed the CH-8 and CH-9 as two doublets, each integrated for one proton at δ 7.76 and 8.03 ppm, respectively. The ^1^H NMR spectrum of compound **17** showed the CH-8,9 and CH-7,10 as two multiplets, each integrated for two protons at δ 2.09–2.21 and 2.42–2.67 ppm, respectively, in addition to the CH-11 as a distorted singlet at δ 7.67 ppm. The spectra also showed other signals at their expected chemical shifts. The ^13^C NMR spectrum of **16** showed three signals at 129.68, 137.56, and 138.17 ppm due to the methine carbons C-9, C-8, and C-3, respectively, and five signals at 90.59, 138.47, 146.96, 158.47, and 164.23 attributed to five quaternary carbons C-3a, C-10a, C-7, C-4, and C-9a, respectively. In addition, the spectra showed signals attributed to carbons of the two phenyl substituents at their expected chemical shifts. Mass spectrum of **14** showed the correct molecular ion peak (M^+●^) which confirmed its molecular weight. MS of **15–17** did not show their molecular ion peaks.

### Physicochemical studies

Since lipophilicity is a significant physicochemical property that determines distribution, bioavailability, metabolic activity, and elimination, the corresponding theoretical C log P values in *n*-octanol buffer were calculated [34].

### Biological evaluation

#### Anti-inflammatory activity

**Fig. 2:**
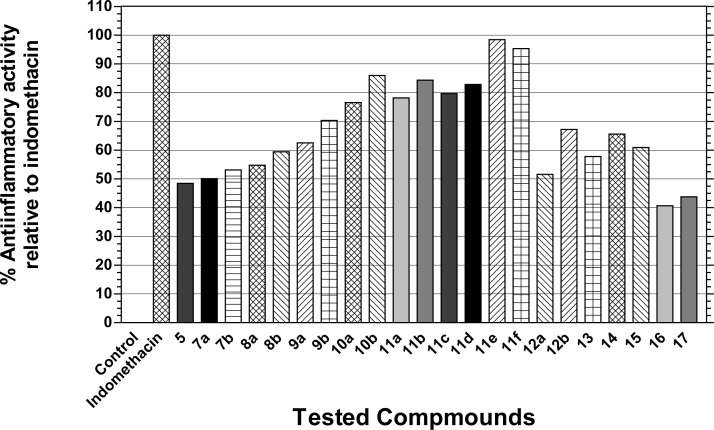
The Anti-inflammatory Effect of the Tested compounds.

The *in vivo* anti-inflammatory effects of the newly synthesized pyrazolopyrimidine derivatives were assessed by utilizing the functional model of carrageenan-induced rat paw edema [35] using indomethacin as the reference drug. Mean changes and the percent increase in paw edema weight of animals pretreated with the tested compounds after 4 h from the induction of inflammation were measured, together with the percent inhibition of induced rat paw edema by the tested compounds. The anti-inflammatory activity (relative potency) of the tested compounds relative to that of indomethacin was also calculated (Table 1) (Fig. 2).

Carrageenan-induced inflammation is a non-specific inflammation resulting from a complex of diverse mediators [36]. It is believed to be biphasic [35], the early phase (1–2 h) involves the release of histamine, serotonin, and bradykinin, and the late phase (3–4 h) is due to the release of prostaglandin-like substances [37–39]. Accordingly, a decrease in the second phase may be attributed to inhibition of cyclooxygenase [39]. This explains the weak inhibitory effect of NSAIDs, such as indomethacin, in the early phase, in contrast to their strong inhibition in the late phase [40]. Therefore, the carrageenan-induced rat paw edema model is conventional, sensitive, and accepted for the screening of newer anti-inflammatory agents [41].

Results listed in Table 1 revealed that the pyrazolopyrimidine derivatives bearing thiazolidinone (**10a,b)** and thiazoline (**11a–f**) moieties exhibited good anti-inflammatory activity (52.23–66.88% edema reduction), while the rest of the tested compounds exhibited moderate activity (28.03–47.77% edema reduction). The reference drug indomethacin induced a 68.15% edema reduction at an equivalent dose as compared to the control group.

Regarding the effect of the electronic nature of the para-substituent on the activity, results revealed that among the thiazolidinone derivatives **10a,b,** compound **10b** with the electron withdrawing chloro group exhibited higher activity (58.60% edema reduction) than the unsubstituted compound **10a** (52.23% edema reduction), corresponding to 85.99% and 76.64% of indomethacin activity, respectively. This relationship between the electronic nature of the substituent and activity holds true in most of the other series.

Among the thiazoline derivatives **11a–c**, having a phenyl ring at 3′-position, compound **11a** (4′-phenyl) and compound **11c** (4′-*p*-chlorophenyl) were nearly equipotent and displayed the same anti-inflammatory activity (53.50% and 54.14% edema reduction, respectively) corresponding to 78.50% and 79.44% of indomethacin activity, respectively, whereas the 4′-p-bromophenyl analog **11b** was slightly more active (57.32% edema reduction) showing 84.11% of indomethacin activity.

Replacing the phenyl ring at the 3′-position of the thiazoline nucleus in **11a–c** with the *p*-chlorophenyl moiety in compounds **11d–f** led, in most cases, to higher percentages of edema reduction. While compound **11d** (4′-phenyl) exhibited considerable anti-inflammatory activity (56.69% edema reduction) corresponding to 83.18% of indomethacin activity, its 4′-*p*-bromophenyl analog **11e** and 4′-*p*-chlorophenyl analog **11f** exhibited the most potent anti-inflammatory activities among the tested compounds with 66.88% and 64.97% edema reduction corresponding to 98.14% and 95.33% of indomethacin activity, respectively. The good anti-inflammatory activity of compounds **11e** and **11f** could be attributed to the presence of a second electron withdrawing bromo or chloro group, respectively, at the para-position of the phenyl ring present at the 4′-position of the thiazoline moiety. Furthermore, since lipophilicity is a significant physicochemical property for anti-inflammatory drugs which determines their distribution in the body and their ability to cross membranes and enter cells [42], the higher lipophilicity value (C log P) of **11e** and **11f** (C log P = 8.23 and 8.08, respectively) supports high activity. Concerning the thiazoline derivatives **11c** and **11d**, lipophilicity does not seem to influence their biological response since C log P of **11c** = C log P of **11d** = 7.37. It is interesting that the potency slightly increased when a *p*-chlorophenyl moiety was incorporated into the 3′-position of the thiazoline nucleus in **11d** (56.69%), rather than at the 4′-position in **11c** (54.14%).

The presence of an imino linkage separating the rigid heterocyclic ring structures from the pyrazolopyrimidine nucleus in compounds **10a,b** and **11a–f** seems to favorably affect their biological responses in comparison to the corresponding thiazolidinone derivatives **8a,b** and dioxopyrimidinethione derivatives **12a,b** lacking such a spacer. Compounds **8a,b** and **12a,b** exhibited moderate anti-inflammatory activity with 37.58%, 40.76%, 35.03%, and 45.86% edema reduction corresponding to 55.14%, 59.81%, 51.40%, and 67.29% of indomethacin activity, respectively. Furthermore, moderate anti-inflammatory activity corresponding to 48.60%, 50.46%, 53.28%, 62.63%, and 70.10% of indomethacin activity was displayed by the 6-methylpyrazolopyrimidine derivatives substituted at the 5-position with an amino (**5**), an arylidene amino (**7a,b**), or an arylthiocarbamoylamino (**9a,b**) function, respectively.

Among the tricyclic pyrazolo[3′,4′:4,5]pyrimido[1,2-*b*]pyridazin-4(1*H*)-one **14–16**, the 7-NH_2_, 8-COOC_2_H_5_ derivative **14** and its 7-OH analog **15** caused 44.59% and 41.40% edema reduction retaining better activity comparable to that of the starting compound **13** (39.49% edema reduction). In contrast, the 7-phenyl analog **16** showed the least anti-inflammatory activity among the tested compounds, with 28.05% edema reduction which is equal to 41.13% of indomethacin activity.

Replacement of the isolated 7-phenyl ring in compound **16** by a cyclohexyl ring fused to the 7- and 8-positions of the pyrazolo[3′,4′:4,5]pyrimido[1,2-*b*]pyridazin-4(1*H*)-one nucleus imparted only slightly higher anti-inflammatory activity for the tetracyclic analog **17** (29.94% edema reduction) comparable to that of compound **16** (28.03% edema reduction).

Results also indicated the correlation between the percent edema reduction by the synthesized bicyclic derivatives **5–13** and lipophilicity theoretically calculated C log P values. Higher activity corresponds to a high C log P value (Table 1). On the other hand, no clear relationship between percent edema reduction and C log P values emerged for the tricyclic **14–16** and tetracyclic **17** derivatives (Table 1).

#### Gastric ulcerogenic activity

Compounds **10b** and **11a–f** exhibiting moderate to potent anti-inflammatory profiles in the pre-mentioned animal models were evaluated for their ulcerogenic potential in rats (Table 2) according to Meshali’s method [43]. The ulcer index was calculated according to Robert’s method [44].

**Fig. 3:**
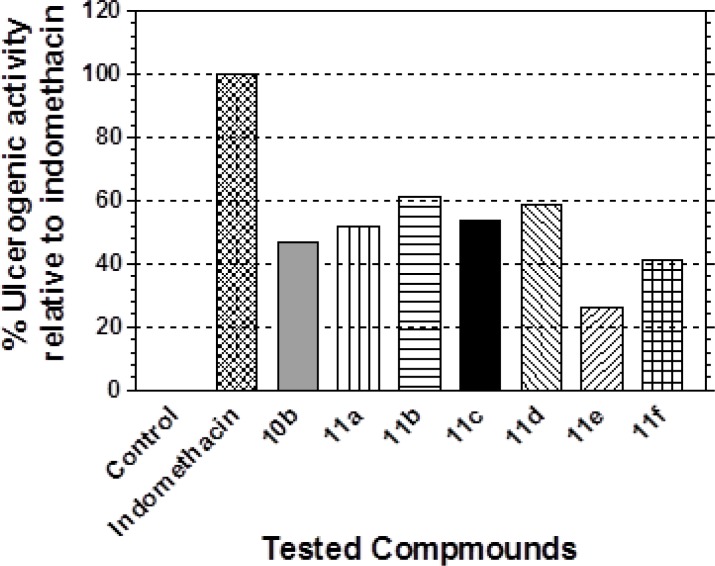
The Ulcerogenic Effect of the Tested compounds.

Results (Table 2) (Fig. 3) indicated that compounds **10b, 11a, 11c,** and **11f** at the oral doses of 10 mg/kg body weight exhibited little gastric ulcerogenic effects, about 41–53% that of indomethacin. Also, they showed better gastrointestinal safety profiles (40–60% ulceration) in the population of test animals when compared to indomethacin, which was found to cause 100% ulceration under the same experimental conditions. In addition, the thiazoline derivative **11e** proved to have very little ulcerogenic effects with the lowest ulcer index corresponding to 26% of indomethacin’s and a superior gastrointestinal safety profile (20% ulceration) in the population of test animals. On the other hand, it should be pointed out that compounds **11b** and **11d** exhibited the highest ulcer index (10.53 and 10.20, respectively) and produced ulceration in 80% of the experimental animals, but their ulcerogenic effect is still much less than that produced by indomethacin (about 61% and 59% that of indomethacin, respectively). These results revealed the advantageously better gastric tolerance of the tested compounds compared to indomethacin.

## Experimental

### Chemistry

Melting points were determined in open glass capillaries on a Stuart melting point apparatus and were uncorrected. The IR spectra were recorded, for potassium bromide discs, ν (cm^−1^), on the Perkin Elmer 1430 spectrophotometer. The ^1^H NMR spectra were determined on Jeol (500 MHz) at the microanalytical unit, Faculty of Science, Alexandria University, using DMSO-*d*_6_ as a solvent and tetramethylsilane (TMS) as internal standard. The chemical shifts are given in ppm δ values. Splitting patterns were designated as follows: s, singlet; d, doublet; t, triplet; and m, multiplet. The ^13^C NMR spectra were determined on Jeol (125 MHz), Faculty of Science, Alexandria University, using TMS as internal standard. Mass spectra were run on a Finnigan mass spectrometer model SSQ/7000 (70 ev), Faculty of Science, Cairo University. Microanalyses were performed at the microanalytical unit, Faculty of Science, Cairo University and at the microanalytical unit, Central lab., Faculty of Pharmacy, Alexandria University. The results of the microanalysis were within ±0.4% of the calculated values. For column chromatography, silica gel (60–200 mesh size), Adwic Laboratory Chemicals, Cairo, Egypt was used. Follow-up of the reactions and checking the homogeneity of the compounds were done by the ascending TLC run on silica gel G (Merck 60)-coated glass plates visualized by iodine vapors. Preparative TLC was performed on 20×20 cm plates coated with 30 g silica gel 60 GF 254 for TLC, Adwic Laboratory Chemicals. A duo-UV lamp (λ 254 nm), Desaga, Heidelberg, Germany was used to find the location of the spots.

Compounds **1**[26], **2**[24, 25], **3**[27], **4**[27], and **6**[27] were prepared according to the reported procedures.

### 5-Amino-6-methyl-1-phenyl-1,5-dihydro-4H-pyrazolo[3,4-d]pyrimidin-4-one (5)

#### Method A: By reaction of hydrazine hydrate with pyrazolooxazine (**4**):

As previously described by Pathak *et al.*[27], compound **5** was prepared in 27% yield (ethanol–chloroform); m.p. 214–215°C, (reported yield 30%, m.p. 215–217°C).

#### Method B: By reaction of hydrazine hydrate with the N-acetylated aminoester (**6**):

A mixture of **6** (2.73 g, 10 mmol) and hydrazine hydrate 99% (10 ml) in ethanol (25 ml) was heated under reflux for 6 h during which the product was partially crystallized out. The reaction mixture was cooled, and the separated product was filtered, washed with ethanol, dried, and crystallized from ethanol-chloroform mixture to yield 2.01 g (86%) of **5** as white crystals, mp: 214–216 °C; IR (KBr, ν, cm^−1^): 3319, 3210, 3112 (NH), 1695 (C=O), 1607, 1567 (C=N), 1536, 1510 (C=C), 1404, 1323 (C–N lactam); ^1^H NMR (500 MHz, DMSO-*d*_6_) δ (ppm): 2.58 (s, 3H, CH_3_), 5.73 (s, 2H, NH_2_, D_2_O exchangeable), 7.34 (t, 1H, *J* = 7.65 Hz, CH-4), 7.48 (t, 2H, *J* = 7.65 Hz, CH-3,5), 7.53 (d, 2H, *J* = 7.65 Hz, CH-2,6), 8.29 (s, 1H, CH-3); ^13^C-NMR (125 MHz, DMSO-*d*_6_) δ (ppm): 23.21 (CH_3_), 96.64 (C-3a), 122.75 (C-2,6), 127.57 (C-4), 129.82 (C-3,5), 136.27 (C-1), 138.50 (C-3), 138.72 (C-7a), 159.70 (C-4), 164.96 (C-6).

The products obtained from both methods were identical as revealed by mp, mixed mp, and superimposability of the IR and ^1^H NMR spectra. However, method B was superior to method A concerning the yield of the prepared compound.

### General procedure for the synthesis of 5-(arylideneamino)-6-methyl-1-phenyl-1,5-diyhdro-4H-pyrazolo[3,4-d]pyrimidin-4-ones (7a,b)

A mixture of **5** (0.24 g, 1 mmol) and benzaldehyde or 4-chlorobenzaldehyde (1 mmol) in glacial acetic acid (5 ml) was heated under reflux for 4h. The reaction mixture was cooled and poured onto crushed ice. The product obtained was filtered, washed with H_2_O, dried, and crystallized from the appropriate solvent.

#### 5-(Benzylideneamino)-6-methyl-1-phenyl-1,5-dihydro-4H-pyrazolo[3,4-d]pyrimidin-4-one (**7a**)

White powder (88%, EtOH); mp: 184–186 °C; IR (KBr, ν cm^−1^): 1697 (C=O), 1613,1572 (C=N), 1536, 1492 (C=C), 1421, 1314 (C–N lactam); ^1^H NMR (500 MHz, DMSO-*d*_6_) δ (ppm): 2.46 (s, 3H, CH_3_), 7.36–7.56 (m, 8H, Ar CH), 7.67 (d, 2H, *J* = 6.8 Hz, Ar” CH-2,6), 8.05 (s, 1H, CH-3), 8.35 (s, 1H, N=C*H*); EI-MS m/z (%): 329 [M^+●^] (absent), 305 (31.6) [M^+●^ – C_2_H_3_, + 3H], 226 (0.3) [M^+●^ – C_6_H_5_-CH=N, + 1H], 185 (100) [M^+●^ – C_6_H_5_-CH=N, – CH_3_-C≡N, + 1H]. Anal. Calcd. for C_19_H_15_N_5_O (329.36): C, 69.29; H, 4.59; N, 21.26. Found: C, 69.03; H, 4.57; N, 21.19.

#### 5-[(4-Chlorobenzylidene)amino]-6-methyl-1-phenyl-1,5-dihydro-4H-pyrazolo[3,4-d]-pyrimidin-4-one (**7b**)

Yellowish white powder (85%, EtOH/CHCl_3_); mp: 195–196 °C; IR (KBr, ν, cm^−1^): 1698 (C=O), 1610, 1569 (C=N), 1534, 1491 (C=C), 1422, 1315 (C–N lactam), 896 (C–Cl); ^1^H NMR (500 MHz, DMSO-*d*_6_) δ (ppm): 2.52 (s, 3H, CH_3_), 7.38 (d, 2H, *J* = 6.8 Hz, Ar” CH-2,6), 7.51 (t, 1H, *J* = 7.6 Hz, Ar’ CH-4), 7.54–7.64 (m, 4H, Ar’ CH-3,5 + Ar” CH-3,5), 7.69 (d, 2H, *J* = 7.6 Hz, Ar’ CH-2,6), 8.36 (s, 1H, CH-3), 8.88 (s, 1H, N=C*H*); EI-MS m/z (%):365 (6.80) [M^+●^ + 2], 363 (21.15) [M^+●^], 315 (28.85) [M^+●^ – Cl, – CH_3_, +2H], 226 (23.08) [M^+●^ – 4-Cl-C_6_H_4_-CH=N, + 1H], 199 (23.08) [M^+●^ – 4-Cl-C_6_H_4_-CH=N, – C_2_H_3_, + 1H], 184 (100) [M^+●^ – 4-Cl-C_6_H_4_-CH=N, – CH_3_-C≡N]. Anal. Calcd. for C_19_H_14_ClN_5_O (363.80): C, 62.73; H, 3.88; N, 19.25. Found: C, 62.60; H, 3.87; N, 19.18.

### General procedure for the synthesis of 6-Methyl-5-(2-aryl-4-oxo-1,3-thiazolidin-3-yl)-1-phenyl-1,5-dihydro-4H-pyrazolo[3,4-d]pyrimidin-4-ones (8a,b)

To a well-stirred suspension of the appropriate Schiff’s base **7a** or **7b** (1 mmol) in dry benzene (50 ml), mercaptoacetic acid (0.11 g, 1.2 mmol) was added. The reaction mixture was refluxed with stirring for 6 h with simultaneous azeotropic removal of water using a Dean–Stark water separator. After cooling, the solution obtained was washed with a saturated solution of sodium hydrogen carbonate (2 × 50 ml), then with water (2 × 50 ml), and dried over anhydrous sodium sulfate. The solvent was evaporated under reduced pressure and the product was crystallized from ethanol.

#### 6-Methyl-5-(4-oxo-2-phenyl-1,3-thiazolidin-3-yl)-1-phenyl-1,5-dihydro-4H-pyrazolo[3,4-d]-pyrimidin-4-one (**8a**)

Pale yellow needles (74%); mp: 179–181 °C; IR (KBr, ν, cm^−1^): 1720 (C=O), 1659 (C=O), 1610, 1522, 1491 (C=N, C=C), 1314 (C–N lactam), 1286, 1177, 1088 (C–S–C); ^1^H NMR (500 MHz, DMSO-*d*_6_) δ (ppm): 2.46 (s, 3H, CH_3_), 3.75 (d, 1H, *J* = 16.0 Hz, CH-5′), 3.88 (d, 1H, *J* = 16.0 Hz, CH-5′), 5.84 (s, 1H, CH-2′), 6.39 (s, 1 H, CH-5′, enol form), 7.35 (t, 1H, *J* = 7.6 Hz, Ar” CH-4), 7.37 (t, 1H, *J* = 7.6 Hz, Ar’ CH-4), 7.43-7.48 (m, 8H, Ar CH), 7.80 (s, 1H, CH-3), 10.10 (s, 1 H, enolic OH, D_2_O exchangeable); EI-MS m/z (%): 403 [M^+●^] (absent), 379 (1.0) [M^+●^ – C_2_H_3_, + 3H], 199 (32.8) [M^+●^ – C_2_H_3_, – 4-oxo-2-phenyl-1,3-thiazolidine, + 1H], 185 (100) [M^+●^ – 4-oxo-2-phenyl-1,3-thiazolidine, – CH_3_-C≡N, + 1H]. Anal. Calcd. for C_21_H_17_N_5_O_2_S (403.46): C, 62.52; H, 4.25; N, 17.36. Found: C, 62.75; H, 4.25; N, 17.43.

#### 5-[2-(4-Chlorophenyl)-4-oxo-1,3-thiazolidin-3-yl]-6-methyl-1-phenyl-1,5-dihydro-4H-pyrazolo[3,4-d]pyrimidin-4-one (**8b**)

Pale yellow amorphous powder (78%); mp: 191–195 °C; IR (KBr, ν, cm^−1^): 1718 (C=O), 1654 (C=O), 1611, 1559, 1491 (C=N, C=C), 1422, 1314 (C–N lactam), 1282, 1086 (C–S–C), 826 (C–Cl); ^1^H NMR (500 MHz, CDCl_3_) δ (ppm): 2.57 (s, 3H, CH_3_), 3.56 (d, 1H, *J* = 16.0 Hz, CH-5′), 3.65 (d, 1H, *J* = 16.0 Hz, CH-5′), 5.56 (s, 1H, CH-2′), 5.75 (s, 1H, CH-5′, enol form), 7.17 (d, 2H, *J* = 8.4 Hz, Ar” CH-2,6), 7.24 (d, 2H, *J* = 8.4 Hz, Ar” CH-3,5), 7.29–7.36 (m, 3H, Ar’ CH-3,4,5), 7.41 (d, 2H, *J* = 7.6 Hz, Ar’ CH-2,6), 7.68 (s, 1H, CH-3), 9.68 (s, 1H, enolic OH, D_2_O exchangeable); ^13^C-NMR (125 MHz, DMSO-*d*_6_) δ (ppm): 22.73 (CH_3_), 62.82 (C-5′), 63.68 (C-2′), 90.58 (C-5′, enol form), 95.52 (C-3a), 124.08 (Ar’ C-2,6), 128.65 (Ar’ C-4), 129.48 (Ar” C-3,5), 129.61 (Ar’ C-3,5), 130.0 (Ar” C-2,6), 131.05 (Ar” C-4), 135.09 (Ar” C-1), 136.41 (Ar’ C-1), 137.47 (C-3), 138.52 (C-7a), 149.58 (C-4), 163.52 (C-6), 170.37 (C-4′), 179.40 (C-4′, enol form); EI-MS m/z (%): 439 [M^+●^ + 2], 437 [M^+●^] (absent), 415 (4.2) and 413 (13.3) [M^+●^ – C_2_H_3_, + 3H], 341 (3.7) and 339 (10.8) [M^+●^ – SCH_2_, – C=O, – C_2_H_3_, + 3H], 226 (8.1) [M^+●^ – 2-(4-chlorophenyl)-4-oxo-1,3-thiazolidine, + 1H], 186 (100) [M^+●^ – 2-(4-chlorophenyl)-4-oxo-1,3-thiazolidine, –CH_3_-C≡N, + 2H]. Anal. Calcd. for C_21_H_16_ClN_5_O_2_S (437.90): C, 57.60; H, 3.68; N, 15.99. Found: C, 57.83; H, 3.69; N, 16.04.

### General procedure for the synthesis of 1-Aryl-6-methyl-5-(arylthiocarbamoyl)- amino-1,5-diyhdro-4H-pyrazolo[3,4-d]pyrimidin-4-one (9a,b)

A solution of **5** (2.41 g, 10 mmol) in ethanol (25 ml) was treated with the selected isothiocyanate derivative (10 mmol) and heated under reflux for 10–12 h. The reaction mixture was concentrated under vacuum and left to cool to room temperature. The deposited product was filtered and crystallized from ethanol.

#### 1-(6-Methyl-4-oxo-1-phenyl-1,4-dihydro-5H-pyrazolo[3,4-d]pyrimidin-5-yl)-3-phenylthiourea (**9a**)

Pale yellow amorphous powder (76%); mp: 145–150 °C; IR (KBr, ν, cm^−1^): 3404, 3303, 3203 (NH), 1644 (C=O), 1615, 1496 br (C=N, C=C, δ NH), 1355 (C–N lactam), 1549, 1257, 1220, 1045 (NCS amide I, II, III and IV mixed vibrational bands, respectively); ^1^H NMR (500 MHz, DMSO-*d*_6_) δ (ppm): 2.47 (s, 3H, CH_3_), 7.12 (t, 1H, *J* = 6.85 Hz, Ar” CH-4), 7.29 (t, 1H, *J* = 7.65 Hz, Ar’ CH-4), 7.37 (t, 2H, *J* = 6.85 Hz, Ar” CH-3,5), 7.44 (t. dist., 2H, Ar’ CH-3,5), 7.51 (d, 2H, *J* = 6.85 Hz, Ar” CH-2,6), 7.53 (d, 2H, *J* = 7.65 Hz, Ar’ CH-2,6), 7.95 (s, 1H, CH-3), 9.55 (s, 1H, *NH*-C_6_H_5_, D_2_O exchangeable), 9.93 (s, 1H, pyrazolopyrimidine-*NH*, D_2_O exchangeable); ^13^C NMR (125 MHz, DMSO-*d*_6_) δ (ppm): 19.50 (CH_3_), 96.28 (C-3a), 123.81 (Ar’ C-2,6), 126.37 (Ar” C-4), 127.85 (Ar’ C-4), 128.43 (Ar” C-2,6), 130.01 (Ar” C3,5 and Ar’ C-3,5), 138.39 (Ar” CH-1), 138.52 (Ar’ C-1), 139.50 (C-3), 139.85 (C-7a), 150.12 (C-4), 164.51 (C-6), 181.98 (C=S); EI-MS m/z (%): 376 [M^+●^] (absent), 318 (1.9) [M^+●^ – S, – C_2_H_3_, + 1H], 302 (0.8) [M^+●^ – S, – CH_3_-C≡N, – H], 287 (0.6) [M^+●^ – C_6_H_5_-NH, + 3H], 216 (10.1) [M^+●^ – C_6_H_5_-NH-CS, - C_2_H_3_, + 3H], 186 (69.3) [M^+●^ – C_6_H_5_-NH-CS-NH, – CH_3_-C≡N, + 2H]. Anal. Calcd. for C_19_H_16_N_6_OS (376.43): C, 60.62; H, 4.28; N, 22.33. Found: C, 60.39; H, 4.27; N, 22.26.

#### 1-(4-Chlorophenyl)-3-(6-methyl-4-oxo-1-phenyl-1,4-dihydro-5H-pyrazolo[3,4-d]pyrimidin-5-yl)thiourea (**9b**)

Yellow microcrystalline powder (88%); mp: 157–160 °C; IR (KBr, ν, cm^−1^): 3318, 3184 (NH), 1672 (C=O), 1627, 1492 (C=N, C=C), 1546 (δ NH), 1306 (C–N lactam), 1598, 1258, 1214, 1048 (NCS amide I, II, III and IV mixed vibrational bands, respectively), 877 (C–Cl); ^1^H NMR (500 MHz, DMSO-*d*_6_) δ (ppm): 2.59 (s, 3H, CH_3_), 7.30 (d, 2H, *J* = 7.6 Hz, Ar” CH-2,6), 7.36 (t, 1H, *J* = 6.9 Hz, Ar’ CH-4), 7.43 (d, 2H, *J* = 7.6 Hz, Ar” CH-3,5), 7.47 (t, 2H, *J* = 6.9 Hz, Ar’ CH-3,5), 7.50 (d, 2H, *J* = 6.9 Hz, Ar’ CH-2,6), 7.94 (s, 1H, CH-3), 9.70 (s, 1H, *NH*-4-Cl-C_6_H_4_, D_2_O exchangeable), 9.97 (s, 1H, pyrazolopyrimidine-*NH*, D_2_O exchangeable); EI-MS m/z (%): 412 (5.7) [M^+●^ + 2], 410 (21.2) [M^+●^], 362 (15.25) [M^+●^ – Cl, – CH_3_, + 2H], 328 (15.25) [M^+●^ – Cl, – CH_3_, – H_2_S, + 2H], 315 (23.73) [M^+●^ – Cl, – C_2_H_3_, – H_2_S, + H], 239 (47.46) [M^+●^ – 4-Cl-C_6_H_4_-N=C=S, – 2H], 226 (23.71) [M^+●^ – 4-Cl-C_6_H_4_-NH-CS-NH, + 1H], 184 (45.76) [M^+●^ – 4-Cl-C_6_H_4_-NH-CS-NH, – CH_3_-C≡N]. Anal. Calcd. for C_19_H_15_ClN_6_OS (410.88): C, 55.54; H, 3.68; N, 20.45. Found: C, 55.76; H, 3.69; N, 20.53.

### General procedure for the synthesis of 5-[3-Aryl-4-oxo-1,3-thiazolidin-2-ylidene)-amino]-6-methyl-1-phenyl-1,5-dihydro-4H-pyrazolo[3,4-d]pyrimidin-4-ones (10a,b)

A solution of the appropriate thiourea derivative **9a** or **9b** (1 mmol) in absolute ethanol (20 ml) was treated with ethyl bromoacetate (0.167 g, 1 mmol) and anhydrous sodium acetate (0.082 g, 1 mmol). The reaction mixture was heated under reflux for 6–8 h, the solvent was evaporated under reduced pressure, and the residue was extracted with chloroform (3×20 ml). The chloroformic layer was washed with water, dried (anhydrous Na_2_SO_4_), and evaporated under vacuum. The products **10a** and **10b** were purified by preparative TLC using C_6_H_6_: EtOAc (8:2, v/v) as developing solvent.

#### 6-Methyl-5-[(4-oxo-3-phenyl-1,3-thiazolidin-2-ylidene)amino]-1-phenyl-1,5-dihydro-4H-pyrazolo[3,4-d]pyrimidin-4-one (**10a**)

White needles (82%, EtOH/CHCl_3_); mp: 264–267 °C; IR (KBr, ν, cm^−1^): 1741 (C=O), 1654 (C=O), 1618, 1595 (C=N), 1559, 1493 (C=C), 1411, 1310 (C–N lactam), 1275, 1071 (C– S–C); ^1^H NMR (500 MHz, DMSO-*d*_6_) δ (ppm): 2.46 (s, 3H, CH_3_), 4.16 (s, 2H, CH-5′), 6.45 (s, 1 H, CH-5′, enol form), 6.86 (d, 2H, *J* = 7.6 Hz, Ar” CH-2,6), 7.10 (t, 1H, *J* = 7.6 Hz, Ar” CH-4), 7.33 (t, 2H, *J* = 7.6 Hz, Ar” CH-3,5), 7.41–7.52 (m, 3H, Ar’ CH-3,4,5), 7.57 (d, 2H, *J* = 8.4 Hz, Ar’ CH-2,6), 8.03 (s, 1H, CH-3), 10.75 (s, 1 H, enolic OH, D_2_O exchangeable); EI-MS m/z (%): 416 [M^+●^] (absent), 392 (18.4) [M^+●^ – C_2_H_3_, + 3H], 300 (1.2) [M^+●^ – C_6_H_5_-NCO, + 3H], 211 (1.2) [M^+●^ – 4-oxo-3-phenyl-1,3-thiazolidin-2-ylideneamino, – CH_3_, + 1H], 186 (100) [M^+●^ – 4-oxo-3-phenyl-1,3-thiazolidin-2-ylideneamino, – CH_3_-C≡N, + 2H]. Anal. Calcd. for C_21_H_16_N_6_O_2_S (416.46): C, 60.56; H, 3.87; N, 20.18. Found: C, 60.35; H, 3.86; N, 20.11.

#### 5-{[3-(4-Chlorophenyl)-4-oxo-1,3-thiazolidin-2-ylidene]amino}-6-methyl-1-phenyl-1,5-dihydro-4H-pyrazolo[3,4-d]pyrimidin-4-one (**10b**)

Pale yellow needles (87%, EtOH); mp: 264–267 °C; IR (KBr, ν, cm^−1^): 1714 (C=O), 1651 (C=O), 1613, 1589 (C=N), 1522, 1494 (C=C), 1411, 1311 (C–N lactam), 1246, 1078 (C– S–C), 820 (C–Cl); ^1^H NMR (500 MHz, CDCl_3_) δ (ppm): 2.45 (s, 3H, CH_3_), 4.03 (s, 2H, CH-5′), 6.22 (s, 1 H, CH-5′, enol form), 7.37 (d, 2H, *J* = 8.4 Hz, Ar” CH-2,6), 7.40 (t, 1H, *J* = 7.6 Hz, Ar’ CH-4), 7.52 (t, 2H, *J* = 7.6 Hz, Ar’ CH-3,5), 7.53 (d, 2H, *J* = 7.6 Hz, Ar’ CH-2,6), 7.58 (d, 2H, *J* = 8.4 Hz, Ar” CH-3,5), 7.73 (s, 1H, CH-3), 10.68 (s, 1 H, enolic OH, D_2_O exchangeable); ^13^C-NMR (125 MHz, DMSO-*d*_6_) δ (ppm): 16.96 (CH_3_), 62.35 (C-5′), 85.88 (C-5′, enol form), 95.47 (C-3a), 123.45 (Ar’ C-2,6), 123.80 (Ar” C-2,6), 129.37 (Ar’ C-4), 130.19 (Ar” C-4), 131.82 (Ar” C-3,5), 132.23 (Ar” C-1), 132.64 (Ar’ C-3,5), 133.26 (Ar’ C-1), 136.43 (C-3), 138.06 (C-7a), 149.08 (C-2′), 154.10 (C-4), 162.32 (C-6), 168.25 (C-4′), 181.17 (C-4′, enol form); EI-MS m/z (%): 452 [M^+●^ + 2], 450 [M^+●^] (absent), 354 (23.6) and 352 (64.0) [M^+●^ – C=O, – SCH_2_, – C_2_H_3_, + 3H], 297 (6.0) [M^+●^ – 4-Cl-C_6_H_4_, – C_2_H_3_, – OH, + 2H], 226 (18.4) [M^+●^ – 3-(4-chlorophenyl)-4-oxo-1,3-thiazolidin-2-ylideneamino, + H], 184 (100) [M^+●^ – 3-(4-chlorophenyl)-4-oxo-1,3-thiazolidin-2-ylideneamino, – CH_3_-C≡N]. Anal. Calcd. for C_21_H_15_ClN_6_O_2_S (450.90): C, 55.94; H, 3.35; N, 18.64. Found: C, 56.14; H, 3.36; N, 18.71.

### General procedure for the synthesis of 5-[3,4-Diaryl-1,3-thiazol-2(3H)-ylidene)-amino]-6-methyl-1-phenyl-1,5-dihydro-4H-pyrazolo[3,4-d]pyrimidin-4-ones (11a–f)

A solution of the appropriate thiourea derivative **9a** or **9b** (1 mmol) in absolute ethanol (20 ml) was treated with the selected phenacyl bromide (1 mmol) and anhydrous sodium acetate (0.082 g, 1 mmol). The reaction mixture was heated under reflux for 8–10 h, partially concentrated and left to cool overnight. The separated product was filtered, washed with aqueous ethanol, dried, and crystallized from ethanol.

#### 5-[(3,4-Diphenyl-1,3-thiazol-2(3H)-ylidene)amino]-6-methyl-1-phenyl-1,5-dihydro-4H-pyrazolo[3,4-d]pyrimidin-4-one (**11a**)

White amorphous powder (80%); mp: 258–260 °C; IR (KBr, ν, cm^−1^): 1616 (C=O), 1590, 1497 (C=N, C=C), 1315 (C–N lactam), 1248, 1178, 1075 (C–S–C); ^1^H NMR (500 MHz, DMSO-*d*_6_) δ (ppm): 2.46 (s, 3H, CH_3_), 6.21 (s, 1H, CH-5′), 6.95 (t, 1H, *J* = 6.8 Hz, Ar” CH-4), 7.31 (d, 2H, *J* = 6.8 Hz, Ar” CH-2,6), 7.38–7.41 (m, 3H, *J* = 7.6 Hz, Ar”‘ CH-3,4,5), 7.53 (t, 1H, *J* = 7.6 Hz, Ar’ CH-4), 7.56–7.59 (m, 8H, Ar CH), 7.73 (s, 1H, CH-3); ^13^C-NMR (125 MHz, DMSO-*d*_6_) δ (ppm): 17.64 (CH_3_), 88.34 (C-5’), 95.29 (C-3a), 118.24 (Ar” C-4), 118.67 (Ar’ C-4), 123.01 (Ar”‘ C-4), 123.78 (Ar” C-2,6), 124.17 (Ar’ C-2,6), 128.83 (Ar”‘ C-2,6), 129.29 (Ar”‘ C-1), 129.48 (Ar’ C-1), 129.66 (Ar”‘ C-3,5), 130.01 (Ar’ C-3,5), 134.29 (Ar” C-1), 136.92 (C-3), 137.14 (C-7a), 137.40 (Ar” C-3,5), 146.55 (C-4’), 157.04 (C-2′), 161.17 (C-4), 164.70 (C-6); EI-MS m/z (%): 476 [M^+●^] (absent), 461 (12.4) [M^+●^ – CH_3_], 318 (100) [M^+●^ – C_6_H_5_, – C_2_HS, – C_2_H_3_, + 3H], 184 (54.7) [M^+●^ – 3,4-diphenylthiazol-2(3*H*)-ylideneamino, – CH_3_-C≡N]. Anal. Calcd. for C_27_H_20_N_6_OS (476.55): C, 68.05; H, 4.23; N, 17.64. Found: C, 67.82; H, 4.23; N, 17.57.

#### 5-{[4-(4-Bromophenyl)-3-phenyl-1,3-thiazol-2(3H)-ylidene]amino}-6-methyl-1-phenyl-1,5-dihydro-4H-pyrazolo[3,4-d]pyrimidin-4-one (**11b**)

Yellow microcrystalline powder (88%); mp: 248–252 °C; IR (KBr, ν, cm^−1^): 1621 (C=O), 1599, 1508 (C=N, C=C), 1398, 1314 (C–N lactam), 1253, 1063 (C–S–C), 693 (C–Br); ^1^H NMR (500 MHz, DMSO-*d*_6_) δ (ppm): 2.58 (s, 3H, CH_3_), 5.73 (s, 1H, CH-5′), 7.31 (d, 2H, *J* = 6.9 Hz, Ar” CH-2,6), 7.35 (d, 2H, *J* = 7.6 Hz, Ar”‘ CH-2,6), 7.38–7.40 (m, 3H, Ar” CH-3,4,5), 7.50 (d, 2H, *J* = 7.6 Hz, Ar”‘ CH-3,5), 7.53 (t. dist., 1H, Ar’ CH-4), 7.63 (t, 2H, *J* = 8.4 Hz, Ar’ CH-3,5), 8.02 (d, 2H, *J* = 8.4 Hz, Ar’ CH-2,6), 8.29 (s, 1H, CH-3); EI-MS m/z (%): 557 [M^+●^ + 2], 555 [M^+●^] (absent), 503 (3.8), 501 (4.1) [M^+●^ – SCH, - CH_3_, + 6H], 318 (20.9) [M^+●^ – 4-Br-C_6_H_4_, - C_2_HS, - C_2_H_3_, + 3H], 261 (5.3) [M^+●^ – 4-Br-C_6_H_4_, - C_6_H_5_-N, - C_2_H, - C_2_H_3_, + 5H], 184 (44.08) [M^+●^ – 4-(4-bromophenyl)-3-phenylthiazol-2(3*H*)-ylideneamino, - CH_3_-C≡N]. Anal. Calcd. for C_27_H_19_BrN_6_OS. ½ H_2_O (564.46): C, 57.45; H, 3.57; N, 14.88. Found: C, 57.56; H, 3.58; N, 14.94.

#### 5-{[4-(4-Chlorophenyl)-3-phenyl-1,3-thiazol-2(3H)-ylidene]amino}-6-methyl-1-phenyl-1,5-dihydro-4H-pyrazolo[3,4-d]pyrimidin-4-one (**11c**)

Pale yellow needles (82%); mp: 258–259 °C; IR (KBr, ν, cm^−1^): 1618 (C=O), 1588, 1498 (C=N, C=C), 1418, 1317 (C–N lactam), 1248, 1073 (C–S–C), 862 (C–Cl); ^1^H NMR (500 MHz, DMSO-*d*_6_) δ (ppm): 2.46 (s, 3H, CH_3_), 6.19 (s, 1H, CH-5′), 6.94 (d, 2H, *J* = 6.8 Hz, Ar” CH-2,6), 6.95 (d, 2H, *J* = 7.6 Hz, Ar”‘ CH-2,6), 7.29–7.32 (m, 3H, Ar” CH-3,4,5), 7.38 (d, 2H, *J* = 7.6 Hz, Ar”‘ CH-3,5), 7.50–7.54 (m, 3H, Ar’ CH-3,4,5), 7.57 (d, 2H, *J* = 6.9 Hz, Ar’ CH-2,6), 7.74 (s, 1H, CH-3); EI-MS m/z (%): 512 (0.39) [M^+●^ + 2], 510 (1.09) [M^+●^], 487 (0.48) and 485 (1.41) [M^+●^ – C_2_H_3_, +2H], 378 (1.09) [M^+●^ – 4-Cl-C_6_H_4_, – C_2_H, + 4H], 318 (24.35) [M^+●^ – 4-Cl-C_6_H_4_, – C_2_HS, – C_2_H_3_, + 3H], 261 (4.02) [M^+●^ – 4-Cl-C_6_H_4_, – C_2_H, – C_6_H_5_-N, – C_2_H_3_, + 5H], 184 (33.71) [M^+●^ – 4-(4-chlorophenyl)-3-phenylthiazol-2(3*H*)-ylideneamino, – CH_3_-C≡N]. Anal. Calcd. for C_27_H_19_ClN_6_OS (511.00): C, 63.46; H, 3.75; N, 16.45. Found: C, 63.52; H, 3.76; N, 16.51.

#### 5-{[3-(4-Chlorophenyl)-4-phenyl-1,3-thiazol-2(3H)-ylidene]amino}-6-methyl-1-phenyl-1,5-dihydro-4H-pyrazolo[3,4-d]pyrimidin-4-one (**11d**)

White crystals (91%); mp: 267–269 °C; IR (KBr, ν, cm^−1^): 1613 (C=O), 1590, 1496 (C=N, C=C), 1375 (C–N lactam), 1247, 1095 (C–S–C), 852 (C–Cl); ^1^H NMR (500 MHz, DMSO-*d*_6_) δ (ppm): 2.75 (s, 3H, CH_3_), 6.21 (s, 1H, CH-5′), 7.37 (d, 2H, *J* = 7.65 Hz, Ar” CH-2,6), 7.38 (d, 2H, *J* = 7.65 Hz, Ar” CH-3,5), 7.40 (t, 1H, *J* = 7.65 Hz, Ar”‘ CH-4), 7.53 (t, 1H, *J* = 7.65 Hz, Ar’ CH-4), 7.56–7.60 (m, 8H, Ar CH), 7.73 (s, 1H, CH-3); EI-MS m/z (%): 512 [M^+●^ + 2], 510 [M^+●^] (absent), 354 (25.50) and 352 (66.30) [M^+●^ – C_6_H_5_, – C_2_HS, – C_2_H_3_, + 3H], 297 (8.90) [M^+●^ – C_6_H_5_, – 4-Cl-C_6_H_4_, – C_2_H_3_, + 2H], 226 (18.10) [M^+●^ – 3-(4-chlorophenyl)-4-phenylthiazol-2(3*H*)-ylideneamino, +1H], 184 (100) [M^+●^ – 3-(4-chlorophenyl)-4-phenylthiazol-2(3*H*)-ylideneamino, – CH_3_-C≡N]. Anal. Calcd. for C_27_H_19_ClN_6_OS (511.00): C, 63.46; H, 3.75; N, 16.45. Found: C, 63.31; H, 3.74; N, 16.41.

#### 5-{[4-(4-Bromophenyl)-3-(4-chlorophenyl)-1,3-thiazol-2(3H)-ylidene]amino}-6-methyl-1-phenyl-1,5-dihydro-4H-pyrazolo[3,4-d]pyrimidin-4-one (**11e**)

White microcrystalline powder (84%); mp: 261–263 °C; IR (KBr, ν, cm^−1^): 1610 (C=O), 1589, 1495 (C=N, C=C), 1412, 1314 (C–N lactam), 1248, 1148, 1099 (C–S–C), 870 (C– Cl), 695 (C–Br); ^1^H NMR (500 MHz, DMSO-*d*_6_) δ (ppm): 2.59 (s, 3H, CH_3_), 6.22 (s, 1H, CH-5′), 7.33 (d, 2H, *J* = 8.45 Hz, Ar” CH-2,6), 7.36 (d, 2H, *J* = 8.45 Hz, Ar” CH-3,5), 7.39 (d, 2H, *J* = 8.45 Hz, Ar”‘ CH-2,6), 7.41 (d, 2H, *J* = 8.45 Hz, Ar”‘ CH-3,5), 7.50 (m, 3H, Ar’ CH-3,4,5), 7.54 (d, 2H, *J* = 8.45 Hz, Ar’ CH-2,6), 8.01 (s, 1H, CH-3H); EI-MS m/z (%): 593 [M^+●^ + 4], 591 [M^+●^ + 2], 589 [M^+●^] (absent), 397 (4.7) and 395 (11.6) [M^+●^ – 4-Br-C_6_H_4_, – CH_3_-C≡N, + 3H], 354 (22.2) and 352 (60.2) [M^+●^ – 4-Br-C_6_H_4_, – C_2_HS, – C_2_H_3_, + 2H], 297 (4.1) [M^+●^ – 4-Br-C_6_H_4_, – 4-Cl-C_6_H_4_, – C_2_H_3_ + 2H], 226 (14.1) [M^+●^ – 4-(4-bromophenyl)-3-(4-chlorophenyl)thiazol-2(3*H*)-ylideneamino, +1H], 184 (100) [M^+●^ – 4-(4-bromophenyl)-3-(4-chlorophenyl)thiazol-2(3*H*)-ylideneamino, – CH_3_-C≡N]. Anal. Calcd. for C_27_H_18_BrClN_6_OS (589.89): C, 54.97; H, 3.08; N, 14.25. Found: C, 54.76; H, 3.07; N, 14.19.

#### 5-{[3,4-Bis(4-chlorophenyl)-1,3-thiazol-2(3H)-ylidene]amino}-6-methyl-1-phenyl-1,5-dihydro-4H-pyrazolo[3,4-d]pyrimidin-4-one (**11f**)

Pale yellow crystals (86%); mp: 272–275 °C; IR (KBr, ν, cm^−1^):1613 (C=O), 1589, 1495 (C=N, C=C), 1412, 1311 (C–N lactam), 1247, 1096, 1078 (C–S–C), 851 (C–Cl); ^1^H NMR (500 MHz, DMSO-*d*_6_) δ (ppm): 2.46 (s, 3H, CH_3_), 6.21 (s, 1H, CH-5′), 7.37 (d, 2H, *J* = 8.0 Hz, Ar” CH-2,6), 7.40 (d, 2H, *J* = 6.8 Hz, Ar”‘ CH-3,5), 7.52 (t, 1H, *J* = 7.6 Hz, Ar’ CH-4), 7.54–7.59 (m, 8H, Ar CH), 7.73 (s, 1H, CH-3); EI-MS m/z (%): 549 [M^+●^ + 4], 547 [M^+●^ + 2], 545 [M^+●^] (absent), 354 (17.6) and 352 (51.8) [M^+●^ – 4-Cl-C_6_H_4_, – C_2_HS, – C_2_H_3_, + 2H], 297 (2.4) [M^+●^ – 2 × 4-Cl-C_6_H_4_, – C_2_H_3_ + 2H], 226 (15.0) [M^+●^ – 3,4-bis(4-chlorophenyl)thiazol-2(3*H*)-ylideneamino, +1H], 198 (2.7) [M^+●^ – 3,4-bis(4-chlorophenyl)thiazol-2(3*H*)-ylideneamino, – C_2_H_3_], 184 (100) [M^+●^ – 3,4-bis(4-chlorophenyl)thiazol-2(3*H*)-ylideneamino, – CH_3_-C≡N]. Anal. Calcd. for C_27_H_18_Cl_2_N_6_OS (545.44): C, 59.45; H, 3.33; N, 15.41. Found: C, 59.32; H, 3.33; N, 15.39.

### General procedure for the synthesis of 1-Aryl-3-(6-methyl-4-oxo-1-phenyl-1,4-dihydro-5H-pyrazolo[3,4-d]pyrimidin-5-yl)-2-thioxodihydropyrimidine-4,6(1H,5H)-diones (12a,b)

A mixture of the appropriate thiourea derivative **9a** or **9b** (15 mmol), malonic acid (2.08 g, 20 mmol), and acetyl chloride (10 ml) was heated for 7 h at 50–55 °C on a water bath, cooled, then poured into ice-cold water (50 ml). The separated product was filtered, washed with water, dried, and purified by preparative TLC using C_6_H_6_ : EtOAc : CHCl_3_ (5:1:5, v/v/v) as developing solvent.

#### 1-(6-Methyl-4-oxo-1-phenyl-1,4-dihydro-5H-pyrazolo[3,4-d]pyrimidin-5-yl)-3-phenyl-2-thioxodihydropyrimidine-4,6(1H,5H)-dione (**12a**)

Yellow crystals (78%, EtOH/CHCl_3_); mp: 242–244 °C; IR (KBr, ν, cm^−1^):1707, 1658 (2 C=O, 4,6-dioxopyrimidine), 1634 (C=O, amide), 1597, 1497 (C=N, C=C), 1402, 1310 (C–N lactam), 1579, 1268, 1164, 1024 (N−C=S amide I, II, III and IV mixed vibrational bands, respectively); ^1^H NMR (500 MHz, DMSO-*d*_6_) δ (ppm): 2.25 (s, 3H, CH_3_), 3.75 (s, 2H, CH-5′), 7.36–7.51 (m, 10 H, Ar CH), 8.03 (s, 1H, CH-3); ^13^C-NMR (125 MHz, DMSO-*d*_6_) δ (ppm): 21.66 (CH_3_), 52.49 (C-5′), 105.87 (C-3a), 121.92 (Ar’ C-2,6), 122.28 (Ar” C-2,6), 123.44 (Ar” C-4), 124.24 (Ar’ C-4), 128.51 (Ar” C-3,5), 129.40 (Ar’ C-3,5), 136.45 (Ar” C-1), 136.99 (Ar’ C-1), 137.33 (C-7a), 138.09 (C-3), 145.16 (C-4), 149.64 (C-6), 151.78 (C-4′), 152.97 (C-6′), 157.43 (C-2′); EI-MS m/z (%): 444 [M^+●^] (absent), 312 (8.2) [M^+●^ – C_6_H_5_-N=C=O, – CH_2_, + 1H], 308 (100) [M^+●^ – C_6_H_5_-N=C=S, – 1H], 273 (30.4) [M^+●^ – C_6_H_5_-N=C=O, – CH_2_-C=O, – CH_3_, + 5H], 144 (10.1) [M^+●^ – 2,3-dihydro-3-phenyl-2-thioxo-4,6(1H,5H)-dioxopyrimidine, – CH_3_-C≡N, – N=C=O, + 2H]. Anal. Calcd. for C_22_H_16_N_6_O_3_S (444.47): C, 59.45; H, 3.63; N, 18.91. Found: C, 59.32; H, 3.63; N, 18.87.

#### 1-(4-Chlorophenyl)-3-(6-methyl-4-oxo-1-phenyl-1,4-dihydro-5H-pyrazolo[3,4-d]pyrimidin-5-yl)-2-thioxodihydropyrimidine-4,6(1H,5H)-dione (**12b**)

Pale yellow amorphous powder (81%. EtOH); mp: 198–201 °C; IR (KBr) ν (cm^−1^): 1701, 1664 (2 C=O, 4,6-dioxopyrimidine), 1640 (C=O, amide), 1578, 1494 (C=N, C=C), 1402, 1317 (C–N lactam), 1544, 1237, 1170, 1018 (N−C=S amide I, II, III and IV mixed vibrational bands, respectively), 833 (C−Cl); ^1^H NMR (500 MHz, DMSO-*d*_6_) δ (ppm): 2.05 (s, 3H, CH_3_), 3.00 (s, 2H, CH-5′), 7.45–7.51 (m, 9H, Ar CH), 7.90 (s, 1H, CH-3); EI-MS m/z (%): 480 [M^+●^ + 2], 478 [M^+●^] (absent), 360 (15.8) [M^+●^ – 4-Cl-C_6_H_4_-N-, + 7H], 296 (11.6) [M^+●^ – 4-Cl-C_6_H_4_-N=C=S, – O, + 3H], 267 (42.1) [M^+●^ – 4-Cl-C_6_H_4_-N=C=S, – CH_2_-C=O], 241 (22.1) [M^+●^ – 4-Cl-C_6_H_4_-N=C=S, – CH_2_-C=O, – C=O, + 2H], 226 (27.4) [M^+●^ – 2,3-dihydro-3-(4-chlorophenyl)-2-thioxo-4,6(1H,5H)-dioxopyrimidine, + 1H], 186 (52.6) [M^+●^ – 2,3-dihydro-3-(4-chlorophenyl)-2-thioxo-4,6(1H,5H)-dioxopyrimidine, – CH_3_-C≡N, + 2H]. Anal. Calcd. for C_22_H_15_ClN_6_O_3_S (478.91): C, 55.17; H, 3.16; N, 17.55. Found: C, 54.94; H, 3.15; N, 17.49.

### 5-Amino-4-oxo-1-phenyl-4,5-dihydro-1H-pyrazolo[3,4-d]pyrimidine-6-carbaldehyde (13)

To a hot solution of **5** (2.41 g, 10 mmol) in dioxane (50 ml), powdered selenium dioxide (2.20 g, 20 mmol) was added portion-wise while stirring. After complete addition, the reaction mixture was refluxed with stirring for 6 h. The precipitated selenium was removed by filtration. The filtrate was evaporated under reduced pressure and the dark yellow residue was treated with cold water (50 ml). The product was extracted with chloroform (3×20 ml), washed with water, dried (anhydrous Na_2_SO_4_), and evaporated to dryness under reduced pressure. The residue was purified by chromatography on a column of silica-gel. Elution with a mixture of C_6_H_6_ : EtOAc : CHCl_3_ (5:1:5, v/v/v) yielded **13** as a pale yellow crystals (1.78 g, 69.8%); mp: 145–146°C; IR (KBr, ν, cm^−1^): 3441, 3322, 3188 (NH), 1720 (HC=O), 1696 (C=O), 1616, 1597 (C=N), 1535, 1500 (C=C), 1550 (δ NH), 1411, 1316 (C–N lactam); ^1^H NMR (500 MHz, CDCl_3_) δ (ppm): 4.24 (s, 2H, NH_2_, D_2_O exchangeable), 7.21 (t, 1H, *J* = 6.2 Hz, Ph CH-4), 7.32 (d, 2H, *J* = 6.2 Hz, Ph CH-3,5), 7.37 (d, 2H, *J* = 6.2 Hz, Ph CH-2,6), 8.48 (s, 1H, CH-3), 9.53 (s, 1H, CHO); ^13^C-NMR (125 MHz, DMSO-*d*_6_) δ (ppm): 95.92 (C-3a), 123.65 (Ph C-2,6), 127.98 (Ph C-4), 129.18 (Ph C-3,5), 138.02 (C-3), 138.97 (C-7a), 144.51 (Ph C-1), 157.39 (C-4), 159.10 (C-6), 172.92 (CHO); EI-MS m/z (%): 255 (52.5) [M^+●^], 226 (12.3) [M^+●^ – CHO], 203 (11.5) [M^+●^ – CHO, – C≡N, + 3H], 185 (9.2) [M^+●^ – CHO, – C≡N, – NH_2_, + 1H], 173 (19.6) [M^+●^ – CHO, – C≡N, – N_2_H_2_, + 3H]. Anal. Calcd. for C_12_H_9_N_5_O_2_ (255.23): C, 56.47; H, 3.55; N, 27.44. Found: C, 56.33; H, 3.56; N, 27.34.

### Ethyl 7-amino-4-oxo-1-phenyl-1,4-dihydropyrazolo[3′,4′:4,5]pyrimido[1,2-b]-pyridazine-8-carboxylate (14)

A mixture of **13** (0.306 g, 1.2 mmol) and ethyl cyanoacetate (0.271 g, 2.4 mmol) in sodium ethoxide solution (20 mg of sodium dissolved in 20 ml of absolute EtOH) was heated under reflux for 6 h. After cooling, the reaction mixture was neutralized with 10% acetic acid (pH 6–7), partially concentrated under reduced pressure, and poured onto crushed ice. The precipitated product was filtered, washed with water, air dried, and crystallized from ethanol to yield **14** as a pale yellow amorphous powder (0.328 g, 78.1%); mp: 155– 156 °C; IR (KBr, ν, cm^−1^): 3437, 3287, 3190 (NH), 1727 (C=O, ester), 1678 (C=O, amide), 1620, 1599, 1501 (C=N, C=C), 1534, (δ NH), 1401, 1317 (C–N lactam), 1280, 1020 (C–O–C); ^1^H NMR (500 MHz, DMSO-*d*_6_) δ (ppm): 1.31 (t, 3H, *J* = 6.9 Hz, CH_2_*CH_3_*), 4.15 (q, 2H, *J* = 6.9 Hz, *CH_2_*CH_3_), 5.06 (s, 2H, NH_2_, D_2_O exchangeable), 7.24 (t, 1H, *J* = 8.4 Hz, Ph CH-4), 7.45 (t, 2H, *J* = 8.4 Hz, Ph CH-3,5), 7.52 (d, *J* = 8.4 Hz, Ph CH-2,6), 7.68 (s, 1H, CH-9), 8.29 (s, 1H, CH-3); ^13^C-NMR (125 MHz, DMSO-*d*_6_) δ (ppm): 15.62 (CH_2_*CH_3_*), 62.50 (*CH_2_*CH_3_), 87.27 (C-3a), 123.73 (Ph C-2,6), 124.07 (Ph C-4), 129.59 (Ph C-3,5), 129.99 (C-9), 137.97 (C-8), 138.38 (Ph C-1), 143.87 (C-3), 144.16 (C-10a), 145.15 (C-7), 145.54 (C-4), 147.44 (C-9a), 148.53 (C=O); EI-MS m/z (%): 350 (10.42) [M^+●^], 298 (25.0) [M^+●^ – NH_2_-C≡N, – CH_3_, +5H], 293 (10.42) [M^+●^ – NH_2_, C_2_H_5_O, + 4H], 269 (20.83) [M^+●^ – NH_2_-C≡N, – C_2_H_5_O, + 6H], 211 (12.5) [M^+●^ – NH_2_-C≡N, – C_2_H_5_, – CO_2_, – C_2_H, +1H], 198 (28.1) [M^+●^ – NH_2_-C≡N, – C_2_H_5_, – CO_2_, – C_3_H], 184 (12.5) [M^+●^ – NH_2_-C≡N, – C_2_H_5_, – CO_2_, – C_3_HN]. Anal. Calcd. for C_17_H_14_N_6_O_3_ (350.33): C, 58.28; H, 4.03; N, 23.99. Found: C, 58.46; H, 4.04; N, 24.07.

### Ethyl 4,7-dioxo-1-phenyl-1,4,6,7-tetrahydropyrazolo[3′,4′:4,5]pyrimido[1,2-b]-pyridazine-8-carboxylate (15)

A mixture of **13** (0.383 g, 1.5 mmol) and diethyl malonate (0.32 g, 2.0 mmol) in sodium ethoxide solution (20 mg of sodium dissolved in 20 ml of absolute EtOH) was heated under reflux for 15 h. The reaction mixture was evaporated under reduced pressure. The resulting residue was dissolved in ice-cold water (10 ml) and acidified (pH 5–6) with acetic acid (10%). The precipitated product was filtered, washed with water, air dried, and crystallized from ethanol to give **15** as pale yellow crystals (0.44 g, 83.5%); mp: 172–173 °C; IR (KBr, ν, cm^−1^): 3187, 3066, 2936 (NH or OH associated), 1711 (C=O, ester), 1649, 1622 (2 x C=O, amide), 1596, 1498 (C=N, C=C), 1545 (δ NH), 1396, 1103 (C–N lactam), 1236, 1023 (C–O–C); ^1^H NMR (500 MHz, DMSO-*d*_6_) δ (ppm): 1.40 (t, 3H, *J* = 6.9 Hz, CH_2_*CH_3_*), 4.07 (q. dist., 2H, *CH_2_*CH_3_), 7.35–7.51 (m, 5H, Ph CH), 7.65 (s, 1H, CH-9), 8.07 (s, 1H, CH-3), 9.60 (s, ½ H, amide NH, D_2_O exchangeable), 12.33 (s, ½ H, imidol OH, D_2_O exchangeable); EI-MS m/z (%): 351 [M^+●^] (absent), 337 (0.66) [M^+●^ – CH_3_, + 1H], 327 (1.11) [M^+●^ – COH, + 5H], 263 (0.27) [M^+●^ – C_2_H_5_, – CO_2_, – OH, + 2H], 226 (2.34) [M^+●^ – C_2_H_5_, – CO_2_, – OH, – C_2_N, + 3H], 198 (35.57) [M^+●^ – C_2_H_5_, – CO_2_, – OH, – C_2_N, – C_2_H], 184 (47.39) [M^+●^ – C_2_H_5_, – CO_2_, – OH, – C_2_N, – C_2_HN]. Anal. Calcd. for C_17_H_13_N_5_O_4_ (351.32): C, 58.12; H, 3.73; N, 19.93. Found: C, 57.94; H, 3.72; N, 19.87.

### 1,7-Diphenylpyrazolo[3′,4′:4,5]pyrimido[1,2-b]pyridazin-4(1H)-one (16)

A mixture of **13** (0.383 g, 1.5 mmol) and acetophenone (0.360 g, 3.0 mmol) in sodium ethoxide solution (20 mg of sodium dissolved in 20 ml of absolute EtOH) was heated under reflux for 8 h. After cooling, the reaction mixture was neutralized (pH 6–7) with acetic acid (10%), partially evaporated under reduced pressure, and poured onto crushed ice. The precipitated product was filtered, washed with water, air dried and crystallized from ethanol to yield **16** as a yellowish brown microcrystalline powder (0.37 g, 72.7%); mp: 118–119 °C; IR (KBr, ν, cm^−1^): 1662 (C=O), 1618, 1598 (C=N), 1532, 1499 (C=C), 1403, 1310 (C–N lactam); ^1^H NMR (500 MHz, CDCl_3_) δ (ppm): 7.35–7.55 (m, 10H, 2 × C_6_H_5_ CH), 7.76 (d, 1H, *J* = 6.9 Hz, CH-8), 8.03 (d, 1H, *J* = 6.9 Hz, CH-9), 8.16 (s, 1H, CH-3); ^13^C-NMR (125 MHz, DMSO-*d*_6_) δ (ppm): 90.59 (C-3a), 121.80 (Ph C-2,6), 123.02 (Ph C-4), 126.96 (Ar’ C-3,5), 127.86 (Ar’ C-2,6), 128.47 (Ph C-3,5), 129.68 (C-9), 130.29 (Ar’ C-4), 131.50 (Ar’ C-1), 137.26 (Ph C-1), 137.56 (C-8), 138.17 (C-3), 138.47 (C-10a), 146.96 (C-7), 158.47 (C-4), 164.23 (C-9a); EI-MS m/z (%): 339 [M^+●^] (absent), 317 (8.46) [M^+●^ – C_2_H_2_, + 4H], 226 (3.96) [M^+●^ – C_6_H_5_, – C_2_HN, + 3H], 211 (1.54) [M^+●^ – C_6_H_5_, – C_3_H_2_N, + H], 184 (69.08) [M^+●^ – C_6_H_5_, – 2 × C_2_HN]. Anal. Calcd. for C_20_H_13_N_5_O (339.35): C, 70.79; H, 3.86; N, 20.64. Found: C, 70.58; H, 3.85; N, 20.57.

### 1-Phenyl-7,8,9,10-tetrahydropyrazolo[3′,4′:4,5]pyrimido[1,2-b]cinnolin-4(1H)-one (17)

A mixture of **13** (0.383 g, 1.5 mmol) and cyclohexanone (0.168 g, 2.0 mmol) in sodium ethoxide solution (20 mg of sodium dissolved in 20 ml of absolute EtOH) was heated under reflux for 4 h. After cooling, the reaction mixture was neutralized (pH 6–7) with acetic acid (10%), partially evaporated under reduced pressure, and poured onto crushed ice. The precipitated product was filtered, washed with water, air dried, and crystallized from ethanol to give **17** as a yellow powder (0.31 g, 65.1%); mp: 142–145 °C; IR (KBr, ν, cm^−1^): 1700 (C=O), 1598, 1500 (C=N, C=C), 1403, 1312 (C–N lactam); ^1^H NMR (500 MHz, DMSO-*d*_6_) δ (ppm): 2.09–2.21 (m, 4H, CH-8,9), 2.42–2.67 (m, 4H, CH-7,10), 7.34–7.52 (m, 5H, Ph CH), 7.67 (s. dist., 1H, CH-11), 8.00 (s, 1H, CH-3); EI-MS m/z (%): 317 [M^+●^] (absent), 313 (13.1) [M^+●^ – 4H], 287 (8.6) [M^+●^ – C_2_H_4_, – 2H], 255 (14.6) [M^+●^ – C_5_H_8_, + 6H], 237 (16.6) [M^+●^ – C_4_H_8_, – C≡N, + 2H], 226 (15.3) [M^+●^ – C_5_H_8_, – C≡N, + 3H], 184 (22.8) [M^+●^ – C_5_H_8_, – C≡N, – C_2_HN]. Anal. Calcd. for C_18_H_15_N_5_O (317.34): C, 68.13; H, 4.76; N, 22.07. Found: C, 68.27; H, 4.76; N, 22.16.

### Physicochemical studies

#### Determination of lipophilicity as C log P

Lipophilicity was theoretically calculated as C log P values in an octanol–water-buffer by the ClogP program of Biobyte. [34].

### Biological evaluation

#### Anti-inflammatory activity

The *in vivo* carrageenan-induced rat paw edema model of inflammation was used to evaluate the anti-inflammatory properties of the newly synthesized compounds according to the previously reported Winter *et al*. method [35].

Adult male albino rats weighing 100–120 g (obtained from the animal house of the department of Physiology, Faculty of Medicine, Alexandria University) were used throughout the work. Animals were randomly divided into groups of six rats each. They were kept in an animal house under standard conditions of light and temperature with free access to food and water *ad libitum* and allowed to be accustomed to their environment for two days before testing.

Indomethacin, used as a reference standard (Khahira for Pharmaceutical and Chemical Industry, Cairo, Egypt), and the test compounds were dissolved in DMSO and were injected intraperitoneally at a dose level of 10 mg/kg body weight. Control animals, on the other hand, were intraperitoneally injected with appropriate volume of DMSO only. After one hour of the above treatment, an inflammatory edema in the right hind paw of all animals was induced by subcutaneous injection of 0.05 ml of freshly prepared solution of 1% carrageenan (λ-Carrageenan Sigma–Aldrich Corp. St. Louis, MO, USA) in sterile normal saline (0.9%), into the subplantar tissue of the paw. An equal volume of saline was injected to the left hind paw and served as internal control for the degree of inflammation in the right hind paw.

Four hours after carrageenan injection, animals were decapitated and both the right and left hind paws were excised at a standard point and weighed. The difference in weight from edema between right and left paws was taken as a measure of the degree of inflammation for each animal. The mean increase in weight of the carrageenan-injected paw over the other paw in each group was measured and the percent increase of edema was calculated. The anti-inflammatory efficacy of the tested compounds was assessed by comparing the change in paw weight in the treated animals with that in control animals (treated with DMSO only) and expressed as a percent inhibition of the edema (percent protection against inflammation), calculated according to the following equation:

% Inhibition of edema=(1-Wt/Wc)×100

where Wt is the mean increase in paw weight in rats treated with the test compounds and Wc is the mean increase in paw weight in the control group. The anti-inflammatory activity of the test compounds relative to that of indomethacin was also determined (Table 2).

#### Gastric ulcerogenic effect

The ulcerogenic effect of the most active compounds **10b** and **11a–f** as well as indomethacin, used as the reference standard, was evaluated according to Meshali’s method [43]. Adult male albino rats weighing between 100 and 120 g were used. Animals were divided into groups of five animals each. Rats were fasted 20 h before drug administration. Water was given *ad libitum*. The control group received the vehicle (1% gum acacia) orally. Other groups received indomethacin or test compounds orally in a dose of 10 mg/kg body weight suspended in 1% gum acacia. Rats were fasted for 2 h, allowed to feed for 2 h, then fasted for another 20 h. Rats were given another two doses on the second and third days. On the fourth day, rats were sacrificed by diethyl ether; the stomach of each rat was removed, opened along the greater curvature, and rinsed with cold 0.9% saline. The stomach was stretched, by pins, on a corkboard and inspected with a 3x magnifying lens for any evidence of mucosal damage. The ulcer index was calculated according to Robert’s method [44]. The number of mucosal damage (red spots) was counted and their severity (ulcerogenic severity) was graded from 0 to 4 according to the following score assignment:

**Table:** 

	Score		Score
Normal (No injury)	0	Slight injury	3
Latent small red spot	1	Severe injury	4
Wide red spot	2		

The following figures were calculated:

–– % Incidence/10 = [number of rats showing ulcer of any grade divided by total number of rats in the group × 100]/10.
–– Average number of ulcers: number of ulcers in the group/total number of rats in the group.
–– Average severity: Σ [each ulcer multiplied by its score of severity]/number of ulcers in the group.
  Ulcer index = the sum of the 3 figures.
  Results are tabulated in Table 2.

### Statistics

In the anti-inflammatory study, data obtained were expressed as the mean ± standard error (SEM). Statistical significance was determined by comparing the values of the test compounds and the standard with those obtained from the control group of animals using One-way ANOVA with Dunnett’s post-test using the GraphPad Prism version 3.00 for Windows (GraphPad Software, San Diego California USA, www.graphpad.com). The difference in results was considered significant if P < 0.001. Results are presented in Table 1.

## Conclusions

In the present study, various substituted pyrazolopyrimidines and pyridazine- as well as tetrahydrocinnoline-fused pyrazolopyrimidines were synthesized and screened for anti-inflammatory and ulcerogenic potential. From the preliminary anti-inflammatory screening results, it could be revealed that the pyrazolopyrimidine derivatives bearing the thiazolidinone (**10b**) and thiazoline (**11a–f**) moieties exhibited good anti-inflammatory activity while the rest of the tested compounds exhibited moderate activity. However, none of the newly synthesized compounds were found to be superior over the reference drug.

Taking the results of the ulcerogenicity study of the most active compounds (**10b** and **11a–f**) into consideration, it could be claimed that compounds **11e** and **11f** presented a promising anti-inflammatory profile with high anti-edematous activity comparable to indomethacin. They caused the highest inhibition of carrageenan-induced paw edema, among the tested compounds with minimal ulcerogenic effects and a good safety margin. In addition, compounds **10b** and **11a–d** achieved the advantage of being much less ulcerogenic despite their slightly lower anti-inflammatory activity as compared to indomethacin. The structure and biological activity relationship of the most active compounds showed that the presence of an electron withdrawing bromo or chloro group at the para-position of the phenyl ring attached either to the 3-position of the thiazolidinone ring (**10b**) or to the 3 and 4-positions of the thiazoline ring (**11a–f**), as well as the presence of an imino linkage separating the rigid 5-membered heterocyclic ring structures from the 5-position of the pyrazolopyrimidine nucleus, seem to be responsible for good biological activity in comparison to compounds **8a** and **8b** lacking such a spacer. Furthermore, results showed that the better biological activity profile of the most active derivatives correlates with the lipophilicity theoretically calculated as the C log P values which might be attributed to better distribution and bioavailability of these derivatives.

Since the GI problems due to NSAIDs continue to be the major impediment to their use in therapeutics, the novel properties of the new anti-inflammatory derivatives prove them to be useful lead molecules for the development of better NSAIDs with a greatly improved therapeutic index.

However, further investigation is needed in order to gain insight into the mechanism of action of the examined compounds.

## Figures and Tables

**Sch. 1.**
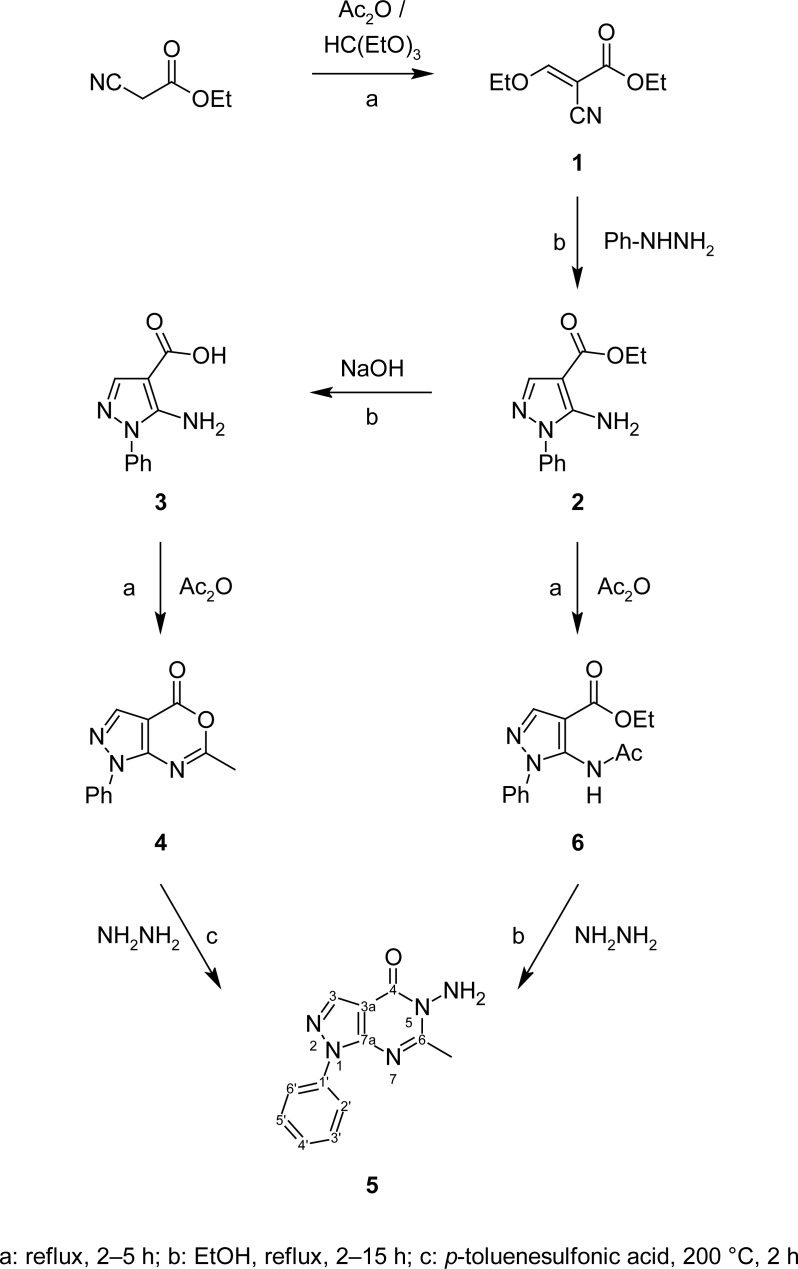
Synthetic routes for the preparation of compound **5**.

**Sch. 2.**
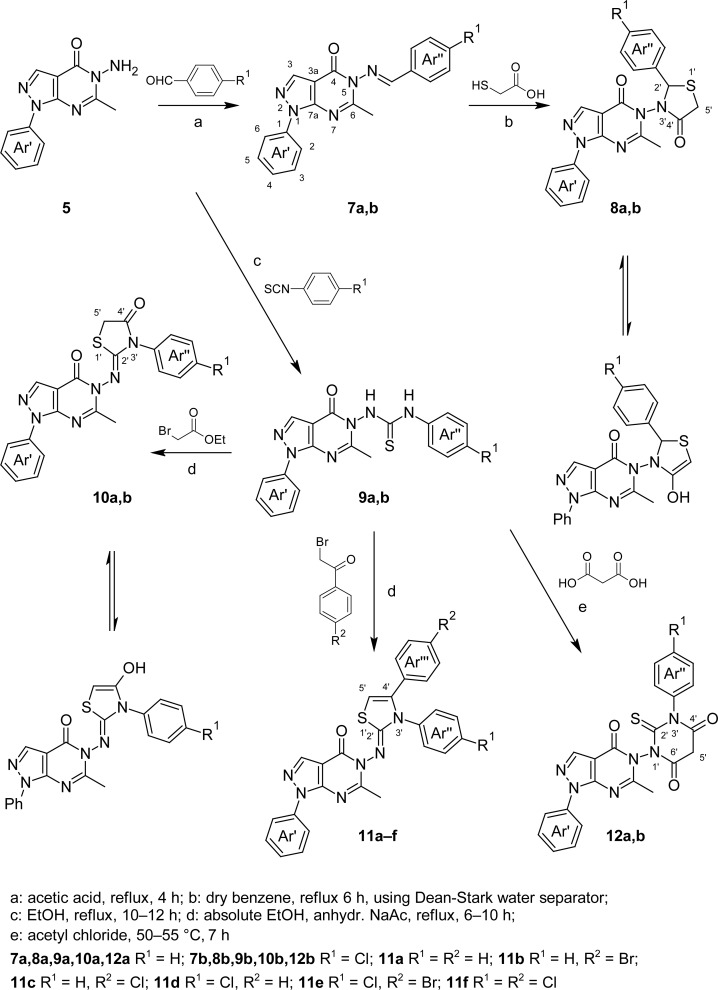
Synthetic routes for the preparation of compounds **7–12.**

**Sch. 3.**
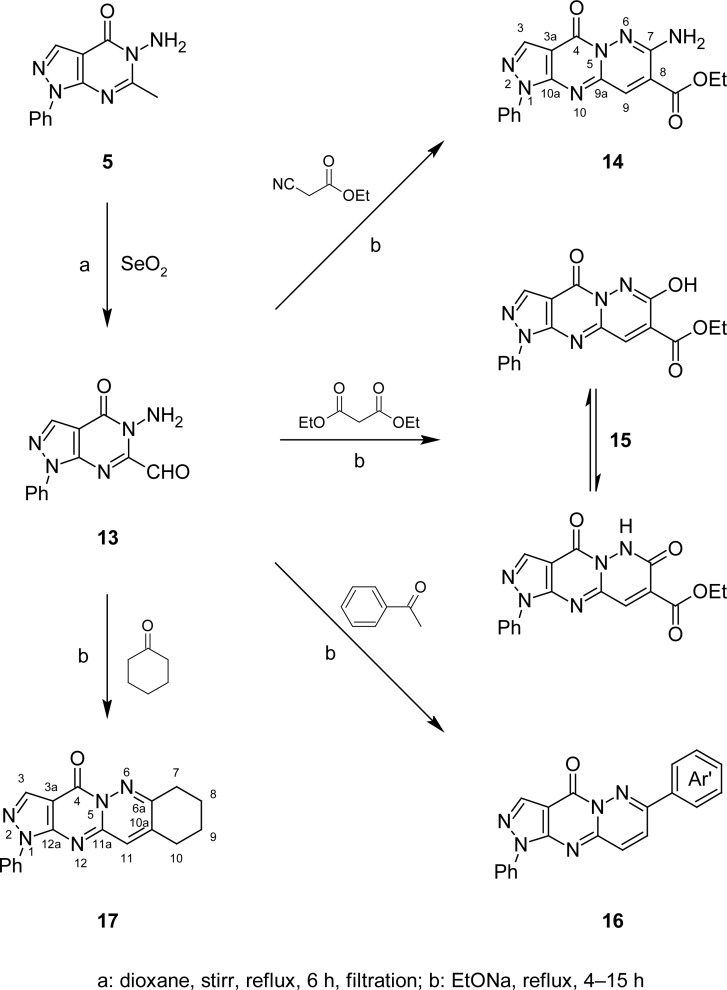
Synthetic routes for the preparation of compounds **13–17.**

**Tab. 1.** Results of anti-inflammatory activity of the tested compounds against carrageenan-induced rat paw edema in rats; Calculated lipophilicity C log P values.

**Comp. No.**	**Mean increase in edema weight ± SEM^a,b^**	**Mean % increase in edema weight ± SEM**	**%Inhibition of Paw edema from control group**	**% Activity relative to indo-methacine**	**C log P**
Control	1.57 ± 0.033	20.84 ± 0.237	–	–	–
Indomethacin	0.50 ± 0.026^*^	6.37 ± 0.366^*^	68.15	100.0	–
5	1.05 ± 0.089^*^	19.82 ± 1.600	33.12	48.60	1.36
7a	1.03 ± 0.033^*^	15.32 ± 0.515^*^	34.39	50.46	2.15
7b	1.00 ± 0.063^*^	15.19 ± 1.400^*^	36.31	53.28	2.87
8a	0.98 ± 0.017^*^	12.48 ± 0.376^*^	37.58	55.14	3.14
8b	0.93 ± 0.042^*^	12.98 ± 0.702^*^	40.76	59.81	3.85
9a	0.90 ± 0.058^*^	12.71 ± 0.748^*^	42.68	62.63	2.09
9b	0.82 ± 0.048^*^	15.71 ± 1.035^*^	47.77	70.10	2.80
10a	0.75 ± 0.022^*^	9.50 ± 0.326^*^	52.23	76.64	2.19
10b	0.65 ± 0.022^*^	8.31 ± 0.430^*^	58.60	85.99	2.91
11a	0.73 ± 0.021^*^	10.54 ± 0.468^*^	53.50	78.50	6.65
11b	0.67 ± 0.049^*^	8.42 ± 0.482^*^	57.32	84.11	7.52
11c	0.72 ± 0.031^*^	10.76 ± 0.457^*^	54.14	79.44	7.37
11d	0.68 ± 0.065^*^	8.73 ± 0.868^*^	56.69	83.18	7.37
11e	0.52 ± 0.031^*^	8.74 ± 0.929^*^	66.88	98.14	8.23
11f	0.55 ± 0.043^*^	9.29 ± 0.961^*^	64.97	95.33	8.08
12a	1.02 ± 0.060^*^	13.56 ± 0.795^*^	35.03	51.40	1.77
12b	0.85 ± 0.043^*^	19.33 ± 0.905	45.86	67.29	2.48
13	0.95 ± 0.043^*^	15.10 ± 1.066^*^	39.49	57.95	1.40
14	0.87 ± 0.076^*^	11.12 ± 1.138^*^	44.59	65.43	1.68
15	0.92 ± 0.060^*^	16.35 ± 1.016^*^	41.40	60.75	1.93
16	1.13 ± 0.067^*^	14.33 ± 0.980^*^	28.03	41.13	2.78
17	1.10 ± 0.052^*^	15.77 ± 0.756 ^*^	29.94	43.93	2.26

a SEM denoted the standard error of the mean.

b Number of animals N = 6 rats.

* Significant difference from control group using Dunnett’s test; p< 0.001.

**Tab. 2.** Ulcerogenic effects of compounds **10b** and **11a–f** in comparison with indomethacin.

**Comp. No.**	**% Incidence/10**	**Average No. of ulcer**	**Average severity**	**Ulcer index**	**% Ulcerogenicity relative to indomethacin**
Control	–	–	–	Nil	–
Indomethacin	10	4.4	2.86	17.26	100.00
10b	6	0.8	1.25	8.05	46.63
11a	6	1.6	1.38	8.98	52.02
11b	8	1.2	1.33	10.53	61.00
11c	6	1.6	1.63	9.23	53.47
11d	8	1.0	1.20	10.20	59.09
11e	2	0.8	1.75	4.55	26.36
11f	4	1.6	1.5	7.10	41.13
